# Short-range translocation by a restriction enzyme motor triggers diffusion along DNA

**DOI:** 10.1038/s41589-023-01504-1

**Published:** 2024-01-02

**Authors:** Martin Göse, Emma E. Magill, Alex Hughes-Games, Steven J. Shaw, Fiona M. Diffin, Tara Rawson, Zsofia Nagy, Ralf Seidel, Mark D. Szczelkun

**Affiliations:** 1 Peter Debye Institute for Soft Matter Physics, Universität Leipzig, Leipzig, Germany; 2 DNA-Protein Interactions Unit, School of Biochemistry, University of Bristol, Bristol, UK

**Keywords:** Single-molecule biophysics, Enzyme mechanisms, DNA

## Abstract

Cleavage of bacteriophage DNA by the Type III restriction-modification enzymes requires long-range interaction between DNA sites. This is facilitated by one-dimensional diffusion (‘DNA sliding’) initiated by ATP hydrolysis catalyzed by a superfamily 2 helicase-like ATPase. Here we combined ultrafast twist measurements based on plasmonic DNA origami nano-rotors with stopped-flow fluorescence and gel-based assays to examine the role(s) of ATP hydrolysis. Our data show that the helicase-like domain has multiple roles. First, this domain stabilizes initial DNA interactions alongside the methyltransferase subunits. Second, it causes environmental changes in the flipped adenine base following hydrolysis of the first ATP. Finally, it remodels nucleoprotein interactions via constrained translocation of a ∼ 5 to 22-bp double stranded DNA loop. Initiation of DNA sliding requires 8–15 bp of DNA downstream of the motor, corresponding to the site of nuclease domain binding. Our data unify previous contradictory communication models for Type III enzymes.

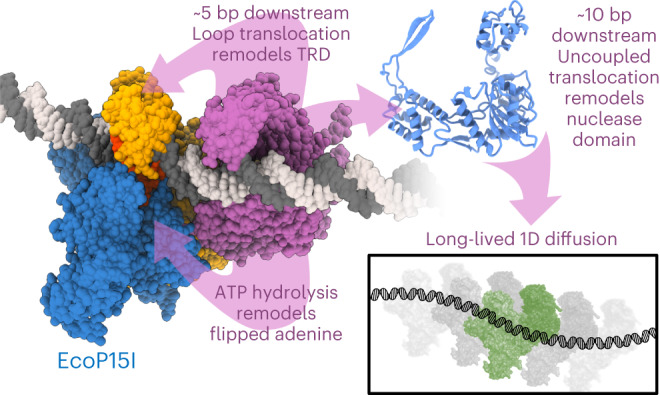

## Abstract (web summary)

Using single-molecule and biochemical methods, Göse et al. demonstrated that the helicase-like ATPase of a Type III restriction enzyme establishes DNA sliding through a sequential series of nucleoprotein remodeling steps driven by DNA translocation.

## Main

**Extended Data Fig. 1:**
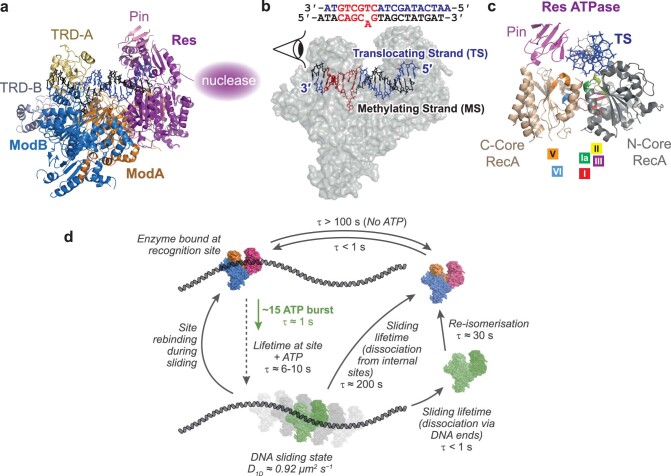
Structural and kinetic features of EcoP15I. **a**, *Escherichia coli* EcoP15I structure (PDB: 4ZCF)^15^ with ModA (orange), Target Recognition Domain-A (TRD-A) (yellow), ModB (blue), TRD-B (light blue), and Res and the Pin domain (magenta) as protein cartoons and DNA (black and blue) as sticks. The nuclease domain was not fully resolved in PDB:4ZCF and is indicated by an oval. **b**, Transparent protein surface view in the same orientation as panel a, with DNA shown as sticks with the translocating strand (TS, blue), methylating strand (MS, black) and recognition site (red), indicated. The eye symbol indicates the direction of the view in panel c. **c**, SF2 helicase-like ATPase domain of Res viewed along the DNA axis (sticks in blue and black) with the N-core RecA (grey), C-core RecA (wheat) and Pin (purple) sub-domains shown as protein cartoons. The helicase-like motifs I (Walker A, red), Ia (green), II (Walker B, yellow), III (purple), V (orange) and VI (light blue) indicated. **d**, Kinetic constants measured during binding, initiation, sliding and re-isomerisation from Refs. ^30,31^. *D*_1D_ is the diffusion coefficient for DNA sliding. DNA is in black; EcoP15I coloured as in panel a, or in green/grey for the isomerised sliding states. With ATP, there is a first phase of ATPase activity of ∼1 s in which ∼10–15 ATPs are consumed. Following a delay, the enzyme leaves the site and enters a long-lived DNA sliding state that retains the enzyme orientation without a requirement for further ATP binding or hydrolysis^29,32^. If a sliding enzyme collides head-to-head with an enzyme bound at a distant site so that the nuclease domains can dimerise, and the second enzyme can hydrolyse ATP^37,44^, DNA cleavage occurs. Collisions with enzymes in incorrect orientations or with other sliding enzymes do not permit cleavage^22,27,32^.

Helicases have a wide range of roles in DNA and RNA metabolism, DNA repair and chromatin rearrangement^1,2^. Although the descriptive enzymatic activity is NTP hydrolysis-dependent separation of two strands of a double-helical polynucleotide, a range of unconventional properties have been discovered for superfamily 2 (SF2) helicases^3^, such as remodeling of DNA–protein complexes (chromatin, stalled polymerases, etc.)^4–7^ or RNA (for example, retinoic acid-inducible gene I (RIG-I)-like receptors)^8^. An example that so far has remained enigmatic is the Type III restriction-modification (RM) enzymes, for example, the *Escherichia coli* enzyme EcoP15I, that protect bacteria and archaea against infections by mobile genetic elements^9–12^. EcoP15I forms a heterooligomeric complex of DNA methyltransferase (Mod) and restriction endonuclease (Res) subunits with a 2:1 Mod:Res stoichiometry (Extended Data Fig. 1a,b)^13–15^. Res contains features characteristic of an SF2 helicase (Extended Data Fig. 1b,c)^16–18^ but lacks strand separation activity. Referred to as a ‘helicase-like ATPase’, EcoP15I hydrolyzes ATP to communicate between pairs of asymmetric restriction sites (5ʹ-CAGCAG-3ʹ) separated on the same DNA by tens to thousands of base pairs, cutting adjacent to one site if both sites are unmethylated and in an inverted-repeat orientation^19–29^. Independently of intersite spacing, fewer than ∼15 ATPs are hydrolyzed by each enzyme for communication and cleavage^30,31^. We previously proposed a long-range communication model based on one-dimensional (1D) diffusion along the DNA contour (‘DNA sliding’) (Extended Data Fig. 1d) that explains the cleavage of sites in both head-to-head and tail-to-tail repeat and overlapping sites^26–35^. Nonetheless, the exact function(s) of the helicase-like ATPase and role(s) for hydrolysis of ∼15 ATPs remained unclear. Here we demonstrate that EcoP15I is a short-range (5–20 bp) double stranded DNA (dsDNA) translocase that disrupts the Mod–DNA interaction, a remodeling activity that is employed to establish the DNA sliding state.

Without ATP, EcoP15I binds its recognition site tightly with a lifetime of >100 s (refs. ^30,31^) and the methylation-target adenine base is flipped out of the helix (5ʹ-CAGCAG-3ʹ; Extended Data Fig. 1a,b)^15,36^. Expanding on the 1D diffusion model, a first putative role for the ATPase activity would be to remodel this nucleoprotein complex, breaking the target recognition domain-A (TRD-A)–DNA contacts and returning the flipped adenine to the helix. Second, the ATPase would need to establish the sliding state (the structural basis of which is unknown). To achieve these two roles, the Res ATPase activity could act as a ‘molecular switch’ to alter the protein–DNA contacts, with the hydrolysis of multiple ATPs most likely reflecting repetitive failed attempts to initiate sliding. Alternatively, hydrolysis of multiple ATPs could be coupled to short-range dsDNA translocation, akin to nucleoprotein remodeling activities such as Mfd acting on stalled RNA polymerases^6,7^. Res motion would produce strain-induced detachment of the TRD-A–DNA contacts, forming the sliding state. Alternative communication models involving unidirectional long-range ATP-dependent dsDNA loop translocation have also been proposed^23,37–40^, mainly based on fast scanning atomic force microscopy (AFM) measurements^40–42^. Although magnetic tweezer measurements appeared to exclude loop translocation as a prerequisite for cleavage^32^, the AFM experiments are consistent with a motor role rather than a switch.

To distinguish between these hypotheses, we established a twist assay with sub-second time resolution based on plasmonic DNA origami nano-rotors^43^ and show that EcoP15I progressively changes DNA twist upon initial ATP-hydrolysis, consistent with loop translocation of ∼5–20 bp of downstream DNA. We furthermore used stopped-flow fluorescence to reveal how translocation remodels the methyltransferase (MTase)–DNA complex. First, changes in the environment of the extrahelical adenine occur upon hydrolysis of the first ATP, interpreted as a reversal of base flipping. Second, TRD movement occurs during translocation, consistent with site release and loop collapse. We also show that forming the DNA sliding state requires at least 10–15 bp of DNA downstream from the helicase initiation site, co-locating with the DNA cleavage site. Based on this, we propose a model where initial short-range dsDNA loop translocation remodels target site binding while downstream DNA translocation is necessary to form a sliding clamp, possibly through remodeling of the nuclease domain, to support communication for hundreds of seconds before dissociation.

## Results

### Short-range dsDNA loop translocation by EcoP15I

**Fig. 1:**
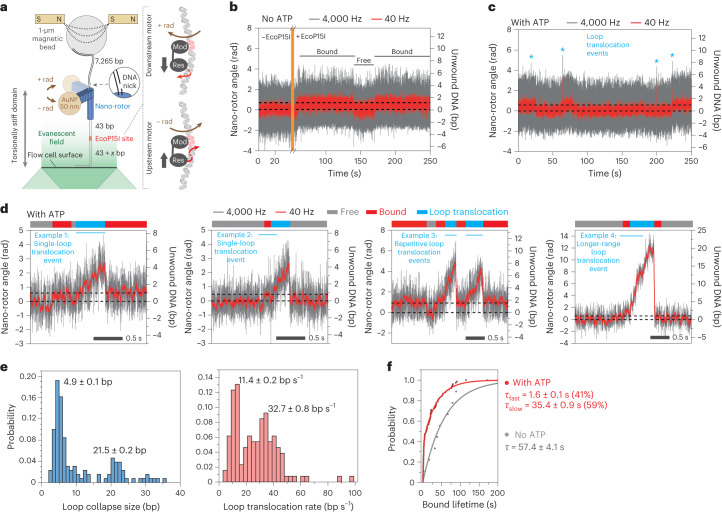
Measurement of DNA loop translocation by EcoP15I. **a**, Left, DNA origami nano-rotor assembly. An EcoP15I target site DNA was attached to the flow cell surface and a nano-rotor consisting of a DNA origami nanostructure with a 56-nm rotor arm and an attached 50-nm AuNP. A paramagnetic bead was attached to a 7.5 kb DNA spacer, allowing force application via two external magnets. Nano-rotor rotations were determined by imaging backscattered light from the AuNP. Right, schematic representation of expected rotation changes upon downstream (top) or upstream (bottom) Res movement, assuming the Mod subunit remains attached and there is no twist compliance in the Res–Mod interactions. **b**, Time trajectory of nano-rotor angular position (gray, at 4,000 Hz; red, after 100-point sliding average ≜ 40 Hz) without EcoP15I and with 4.66 nM EcoP15I. A reversible positive rotational shift of 0.60 ± 0.18 rad (1.0 ± 0.3 bp) is observed with EcoP15I, which we identified as the ‘DNA-bound’ state. **c**, Time trajectory of the nano-rotor angular position with EcoP15I and ATP. In addition to free and bound states, sawtooth-like loop translocation events were also detected (blue asterisks). **d**, Representative examples of different loop translocation events. Bars identify different EcoP15I–DNA interaction states (gray, free state; red, bound state; blue, loop translocation). Loop translocation occurred as short-range single events terminating in a bound (example 1) or free (example 2) state, or clustered short-range events (example 3) and long-range events (example 4). **e**, Maximum loop size and loop translocation rate exhibiting bimodal distributions, with mean values of: 4.9 ± 0.1 bp and 21.5 ± 0.2 bp for length and 11.4 ± 0.2 bp s^−1^ and 32.7 ± 0.8 bp s^−1^ for translocation rate (*n* = 130; errors, s.e.). **f**, Probability distribution of bound state lifetime without ATP (gray, *n* = 9) and with ATP (red, *n* = 76). Average lifetimes of 57.4 ± 4.1 s (without ATP) and 21.5 s (with ATP, 41% at 1.6 ± 0.1 s and 59% at 35.4 ± 0.9 s) were obtained by single-exponential (without ATP) or double-exponential (with ATP) curve fitting (errors, s.e.).

Stepwise EcoP15I translocation would cause the helicase-like motor to follow the DNA helical pitch. Because Res does not release the Mod dimer^13,14^, translocation would form a short, strained DNA loop between the motor domain and TRD-A. Based on the EcoP15I–DNA structure and the properties of SF2 helicases, the 3ʹ–5ʹ strand was designated as the translocating strand (TS) and the complementary strand as the methylating strand (MS) (Extended Data Fig. 1b)^15^. The 3ʹ–5ʹ tracking typical of an SF2 helicase^3^ would move Res downstream (left to right as drawn in Extended Data Fig. 1a,b), pumping DNA into a constrained loop with reduced twist. If Res were to move upstream (right to left) the loop would shorten and become overtwisted. To measure DNA topology changes associated with initial ATPase activity, we developed a nano-rotor approach (Fig. 1a). In brief, we tethered linear DNA between a coverslip and a paramagnetic particle and applied a force of 3 pN to minimize random motion. The DNA contained the following: an EcoP15I site with 43 bp upstream and a variable length sequence (43 + *x* bp) downstream (where *x* is the first randomly introduced surface attachment point; Methods); a plasmonic DNA nano-rotor comprising a DNA origami rotor arm and a 50-nm gold nanoparticle (AuNP)^43^; and a ∼ 7.2-kb DNA spacer including a DNA nick. The site-containing DNA section, bottom attachment and nano-rotor represent a torsionally stiff domain (Fig. 1a). Changes in the linking number of this domain resulting from loop translocation directly promoted nano-rotor rotation because the nick above prevented the generation of any antagonistic torsion. By monitoring the AuNP at 4,000 Hz, ultrafast DNA twist changes could be observed with a spatiotemporal resolution of 1 bp per 80 ms (signal-to-noise ratio of 3; Supplementary Fig. 1). Downstream loop translocation would produce a positive change in angular position while upstream motion would produce a negative change (Fig. 1a). Additionally, any changes in DNA topology associated with protein binding could be inferred.

**Extended Data Fig. 2:**
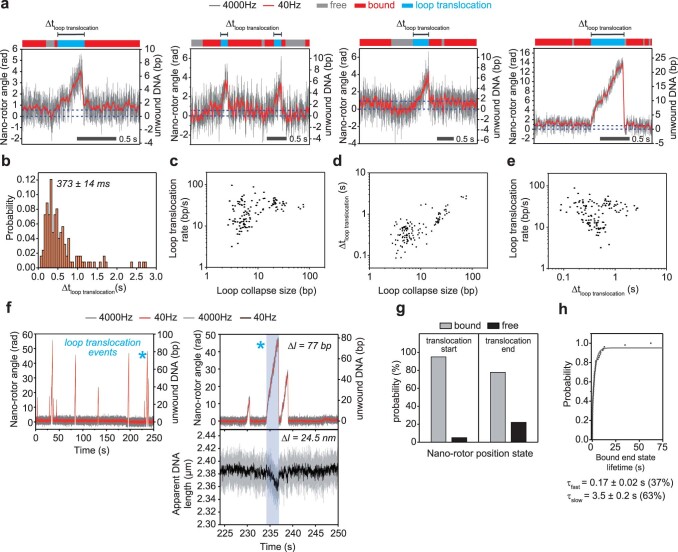
DNA loop translocation properties. **a**, Further examples of translocation events measured with the nano-rotor assay (grey, at 4000 Hz; red, after 100-point sliding average ≜40 Hz) after the addition of 4.66 nM EcoP15I. **b**, Distribution of the loop translocation time (Δt_loop translocation_) with a mean time of 373 ± 14 ms (*n* = 130, errors s.e.). **c-e**, Plots of the loop translocation rate vs. loop collapse size (panel c), Δt_loop translocation_ vs loop collapse size (panel d) and the loop translocation rate vs Δt_loop translocation_ (panel e) plotted for individual events. The loop translocation time increased with the loop collapse size, consistent with a constant stepwise translocation rate, whereas the other parameters did not follow a trend. **f**, (*left panel*) Time trajectory of the angular position of the nano-rotor including long loop translocation events with loop collapse sizes of >40 bp. Asterisk marks the event shown in the right panel. (*right panel*) Trajectory of the angular position of the nano-rotor for the long loop translocation event shown together with the length of the DNA construct revealing the DNA length reduction upon loop translocation. The loop collapse size of ∼77 bp matches with the apparent DNA length reduction of 24.5 nm (∼72 bp) assuming helix translocation with 1 bp ≈ 0.34 nm. **g**, Probabilities that loop translocation events started and ended either in the free or bound state (*n* = 113). Only 5% of loop translocation events started in the free state and 95% in the bound state. 77% of loop translocation events terminated in a bound state and 23% in the free state. **h**, The bound state after loop translocation exhibited a bi-exponential lifetime distribution with an average lifetime of ∼2.3s (37%: 0.17 ± 0.02 s and 63%: 3.5 ± 0.2 s; *n* = 54, errors, s.e.).

Without protein, the angular fluctuation was 0.35 ± 0.04 rad (filtered at 40 Hz). With EcoP15I but without ATP we observed two states that differed by 0.60 ± 0.18 rad (1.0 ± 0.3 bp for one turn every 10.5 bp) (Fig. 1b). EcoP15I-DNA association results in adenine base flipping and DNA bending^15,36^, so we interpret these states as ‘free DNA’ and ‘protein-bound DNA’. With ATP, the two states remained but we additionally observed transient sawtooth-like twist changes towards positive angular displacements: a linear DNA twist increase followed by spontaneous loop release and loss of twist (Fig. 1c,d and Extended Data Fig. 2a), consistent with downstream DNA loop translocation. In 95% of cases, these events were preceded by a clear DNA-bound state, while in 5% of cases binding and initiation were closely spaced, such that unambiguous assignment of a DNA-bound state before translocation was not possible (Fig. 1d and Extended Data Fig. 2g). The end of a loop translocation event defines a ‘loop collapse’, with an associated loop time and distance. Loop collapse could be due to helicase dissociation with TRDs still bound (that is, motor ‘processivity’), TRD dissociation (initiating sliding) or complete complex dissociation from the DNA. None of these events were detected using a nonspecific DNA (Supplementary Fig. 2).

Loop translocation events included the following (Fig. 1d and Extended Data Fig. 2a): (1) short-range loop translocation events (average of 5 bp) that ended in the DNA-bound state (example 1); (2) short-range loop translocation events that ended in the free DNA state (example 2); (3) clustered short-range events, which might have resulted from the same protein molecule undertaking repeated initiation events (these could be events that failed to fully dissociate from the site or where a sliding enzyme immediately re-bound the site and had to re-initiate^30^; example 3) and; (4) longer-range loop translocation events (average of >20 bp) that were observed as both isolated (example 4) and clustered events (Extended Data Fig. 2f). In a few examples of events >40 bp, twist changes were sufficient to reduce the apparent DNA length registered from paramagnetic particle tracking (Extended Data Fig. 2f). We suggest that these infrequent (∼3%) larger looping events resulted from a failure of TRD-A to dissociate, followed by continued translocation, and these events help to explain the loop translocation observed by fast scanning AFM^41,42^. Given that we found these to be rare events, there could be an enzyme subpopulation that always forms larger loops (static enzyme disorder) or occasional reversible conformational transitions to produce states that only form larger loops (dynamic enzyme disorder).

The loop size and translocation rate were both bimodally distributed, with sizes of 4.9 ± 0.1 bp or 21.5 ± 0.2 bp and rates of either 11.4 ± 0.2 bp s^-1^ or 32.7 ± 0.8 bp s^–1^ (Fig. 1e), again suggesting enzyme disorder. Although direct dependence between the two parameters was not observed, events with maximum loop sizes of >12 bp exclusively exhibited the faster loop translocation rate (Extended Data Fig. 2c). The average loop translocation lifetime was 370 ± 20 ms and increased with loop size but did not depend on the observed rate (Extended Data Fig. 2d,e). ATP also influenced the average ‘bound’-state lifetime: without ATP, the bound-state lifetime was 57.4 ± 4.1 s, whereas with ATP we observed a reduced average lifetime of ∼21.5 s as well as a bi-exponential distribution (41% at 1.6 ± 0.1 s and 59% at 35.4 ± 0.9 s) (Fig. 1f and Supplementary Fig. 3). Furthermore, we observed in most events (77%) a clear bound state following loop translocation (Extended Data Fig. 2g) with an average ‘bound’ lifetime of ∼2.3 s (37% at 0.17 ± 0.02 s and 63% at 3.5 ± 0.2 s) (Extended Data Fig. 2h). We suggest that the remodeled state has a similar effect on DNA twist as the pre-bound state, but it may not necessarily represent the same structure.

### Conformation changes during DNA translocation by EcoP15I

**Fig. 2:**
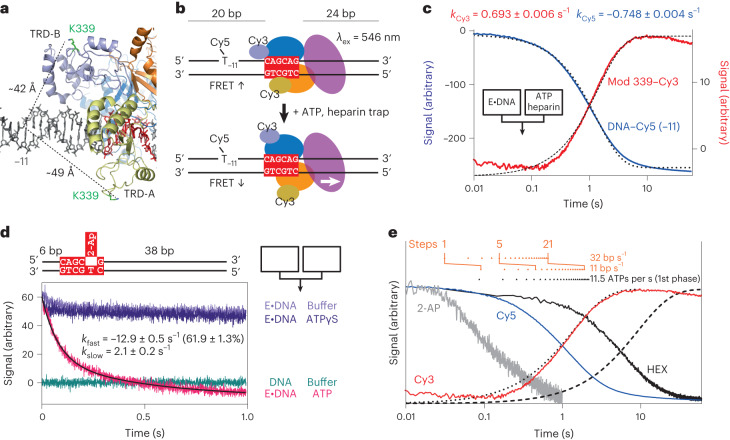
Nucleoprotein conformation changes during the first ATPase burst. **a**, Cropped view of the structure from ref. ^15^. Protein domains are shown as cartoons, colored as in Extended Data Fig. 1a, and DNA is shown as sticks in black and red. The K339 residues from both TRDs are shown as green sticks, with dotted lines indicating the approximate distance to the major groove of the base −11 bp from the recognition site (red). **b**, Cartoon of the oligoduplex used in the FRET assay. The MS was labeled at the dT position −11 with Cy5, and K339 was labeled with Cy3. Movement of the TRDs reduces the FRET. **c**, Stopped-flow fluorescence measurements of a pre-formed EcoP15I 339–Cy3 and Cy5–oligoduplex (E·DNA) complex mixed with ATP and heparin trap. Cy3 (red) and Cy5 (blue) emission fluorescence signals were fitted to single exponentials (dotted lines) to give the rate constants indicated (errors, s.e.m.). **d**, Cartoon of the oligoduplex used in the 2-aminopurine (2-AP) assay. 2-Aminopurine emission signals are shown for stopped-flow fluorescence measurements mixed according to the color key (right). The ATP trace was fitted to a double exponential (black line) to give the rate constants indicated (errors, s.e.m.). **e**, Comparison of EcoP15I kinetics. Cy5 (blue) and Cy3 (red) FRET data shown are from **c**; 2-aminopurine data (gray) shown are from **d**; hexachlorofluoroscein (HEX) anisotropy DNA dissociation data (black) using substrate 38/38 shown are from Supplementary Fig. 8; First phase ATPase burst kinetics (black dotted line) and second phase ATPase kinetics (dashed line) using substrate 38/38 from Supplementary Fig. 9. Cumulative ATP hydrolysis steps for the first phase are also shown as black triangles; cumulative stepping events calculated from the fast and slow translocation rates in Fig. 1e are shown as orange triangles. The first, fifth and 21st steps are indicated.

**Extended Data Fig. 3:**
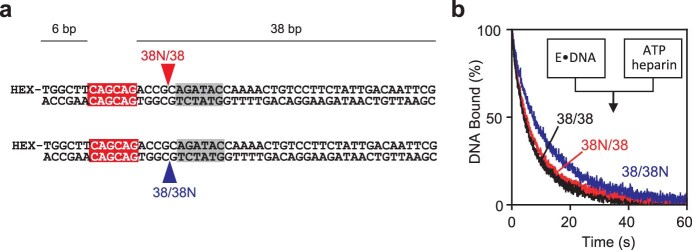
A nick in either strand of the translocated loop does not significantly affect ATP-dependent DNA dissociation. **a**, Nicked oligoduplex substrates. 38N/38 (upper), nicked on the MS 4 bp downstream of the recognition site, was made by annealing the DNA oligonucleotides Nick3_Fwd_Hex, N35_Fwd_Phos and 38_Rev (Supplementary Tables 5, 6). 38/38N (lower), nicked on the TS 4 bp downstream of the recognition site, was made by annealing 38_Fwd_Hex, N23_Rev and N15_Rev_Phos (Supplementary Tables 5, 6). **b**, Release of prebound enzyme from its target site with ATP and heparin measured using stopped flow fluorescence anisotropy. Data is the average of two independent repeats.

We used rapid-mixing stopped-flow fluorescence spectroscopy to further explore how short-range translocation influences both EcoP15I and DNA conformations. To address the remodeling of DNA-TRD contacts, we labeled Mod at K339 with Cy3 and labeled an upstream oligoduplex position with Cy5, giving similar spacings between K339 in TRD-A or TRD-B in the bound state (Fig. 2a,b and Supplementary Fig. 4a–d). The labeled protein had comparable DNA cleavage and loop translocation activities to the wild type (Supplementary Figs. 4e and 5). Rapid mixing of labeled protein with labeled DNA produced anticorrelated changes in Cy3 and Cy5 emission fluorescence consistent with Förster resonance energy transfer (FRET) upon binding (Supplementary Fig. 6a). Rapid mixing of the prebound high-FRET state with ATP and heparin (to trap dissociated protein) produced anticorrelated Cy3 and Cy5 emission changes that returned to free state values (Fig. 2c), although some of the Cy3 signal came from heparin quenching (Supplementary Fig. 6b,c). The data are consistent with TRD-A and/or TRD-B motion on a timescale (∼1.4 s) equivalent to the ATPase burst and to the loop translocation lifetime (Extended Data Fig. 2b). We ruled out the possibility that torsional strain drives remodeling as nicks in the initial loop region did not reduce the DNA dissociation rate (Extended Data Fig. 3).

To address base flipping, we substituted the target adenine with 2-aminopurine in an oligoduplex (Fig. 2d). As expected^36^, DNA association produced a fluorescence increase and a blue shift in the maximum emission wavelength that we interpreted as a change in fluorophore environment consistent with base flipping (Supplementary Fig. 7). Rapid mixing of the pre-bound high-fluorescence/flipped state with ATP produced a fast, biexponential decrease in fluorescence that was not observed with the nonhydrolyzable ATPγS analog which did not support rapid DNA dissociation or sliding (Fig. 2d). We interpret this as being an ATP hydrolysis-triggered environment change of the flipped adenine before TRD remodeling, with the base possibly re-flipping to facilitate site dissociation.

We compared these data with other kinetics measured using oligoduplexes (Fig. 2e), including enzyme dissociation measured by anisotropy of 6-hexachlorofluoroscein-labeled DNA (Supplementary Fig. 8)^30,31^ and ATP hydrolysis quantified by phosphate binding shown as fitted exponential profiles of the first (fast) and second (slow) phases (Supplementary Fig. 9)^31^. Also plotted were base pair steps estimated from fast and slow translocation events (Fig. 1). The first ATP hydrolyzed produced a change in 2-aminopurine fluorescence. The correspondence between the first-phase ATPase kinetics and Cy3 and Cy5 fluorescence changes suggests that multiple translocase steps are needed to remodel the TRDs. There is then a delay before DNA dissociation during which further ATP hydrolysis occurs.

The correspondence between the TRD FRET, ATPase and motor kinetics (Fig. 2e) is consistent with loop translocation producing strain that detaches the DNA TRD contacts. We tested this by measuring loop translocation at a higher stretching force (6 pN; Supplementary Fig. 10); loop size, translocation rate and average loop translocation lifetime distributions were similar to those at 3 pN. Using a combined optical tweezers and confocal scanning fluorescence microscope approach to follow binding and sliding by quantum dot-labeled EcoP15I (ref. ^30^), sliding initiation times were also similar with stretching forces of either 1.5 pN or 15 pN (Supplementary Fig. 11a–c). Furthermore, we did not observe any impact of force on DNA cleavage rates^32^. Force insensitivity may be due to the 1-bp step size of dsDNA translocating helicase-like motors and/or due to an internal allosteric coupling of the remodeling complex that is shielded from external stress. We additionally could not stimulate detachment of the TRD–DNA contacts and induction of sliding by simply increasing the DNA stretching force in the absence of ATP hydrolysis (Supplementary Fig. 11d).

### Roles for downstream DNA in establishing DNA sliding

**Fig. 3:**
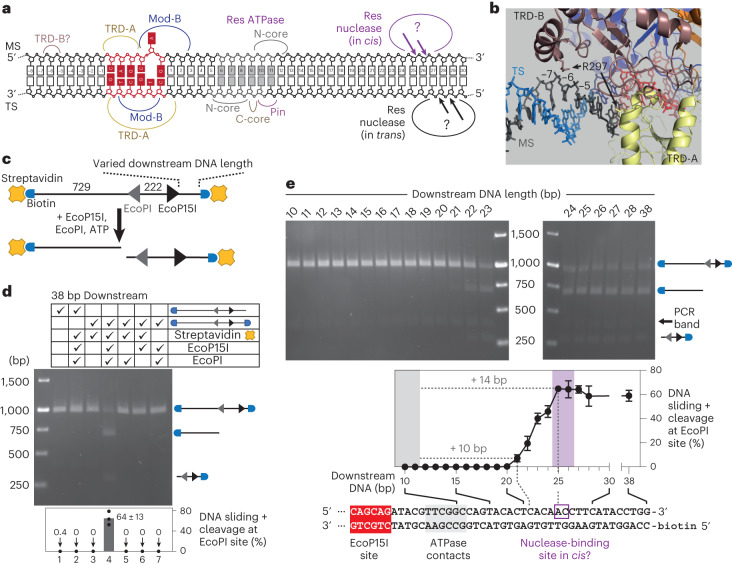
Effect of upstream and downstream DNA on DNA sliding and cleavage. **a**, Locations of protein–DNA contacts (curved lines) from Protein Data Bank (PDB) 4ZCF (ref. ^15^). Nuclease contacts at the DNA cleavage sites (arrows)^50^ are not known. TRD-B upstream contacts are defined in **b**. **b**, Cropped view of PDB 4ZCF with the upstream DNA extended using B-form DNA (PDB 1BNA)^49^. Proteins are shown as cartoons, R297 in each TRD is shown as a stick, and DNA is shown as sticks (MS gray; TS, blue; recognition site, red). **c**, DNA substrate design with sites for EcoP15I (5ʹ-CAGCAG-3ʹ top strand sequence, black) and EcoPI (5ʹ-GGTCT-3ʹ top strand sequence, gray) in tail-to-tail orientation. The distance upstream of the EcoP15I site was varied from 10 to 38 bp. DNA ends were labeled with biotin (blue) and capped with streptavidin (orange). **d**, DNA labeled with biotin at one or both ends was mixed with streptavidin, EcoP15I or EcoPI as indicated. Reactions were quenched after 1 h and DNA was separated by agarose gel electrophoresis (representative gel; *n* = 3 repeats). Mean EcoPI site cleavage was quantified from *n* = 3 repeats (error bars, s.d.). Cleavage at the EcoP15I site was not possible. **e**, Cleavage reactions were carried out for 1 h using DNA capped at both ends with varying downstream DNA lengths as indicated. DNA was separated by agarose gel electrophoresis (representative gel; *n* = 3 repeats) and mean DNA cleavage was quantified from *n* = 3 repeats (error bars, s.d.).

We explored whether continued downstream translocation following loop collapse might be required to establish the sliding state. From the EcoP15I-DNA structure^15^, TRD-A and Mod-B interact with the target sequence and modified adenine, respectively, while initial ATPase contacts are 6–12 bp downstream on both strands (Fig. 3a). The nuclease domain cuts the MS 25–26 bp downstream, although exact contacts are unknown. It has been suggested^44^ that 17 bp of downstream DNA are needed for cleavage in *trans* between DNA oligoduplexes, but it is unclear whether this requires the sliding conformation. By extending the upstream DNA in the cocrystal structure^15^, we noted that TRD-B R297 could contact the MS backbone 5–6 bp upstream (Fig. 3b). However, using a band-shift assay to quantify DNA binding, similar dissociation constant *(K*_d_) values were measured with 2 bp or 10 bp of upstream DNA (Supplementary Fig. 12), suggesting that TRD-B R297 is not vital. This is consistent with previous assays that showed DNA cleavage with 2 bp of upstream DNA^44^.

**Extended Data Fig. 4:**
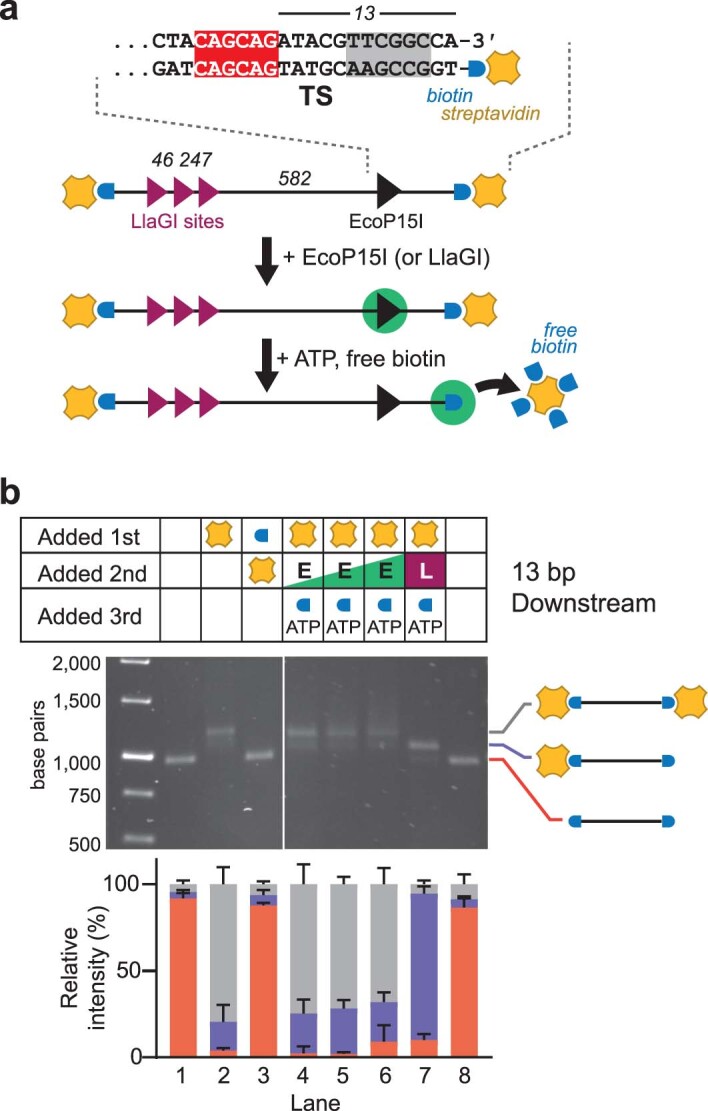
EcoP15I cannot displace streptavidin from biotin-labelled DNA despite its translocase activity. **a**, DNA substrate (Fig. 3c), with a single EcoP15I site 13 bp from the DNA end and with three sites for the Type ISP restriction enzyme LlaGI (5ʹ-CTnGAYG-3ʹ) in direct repeat (purple) where arrowheads indicate the direction of ATP-dependent LlaGI translocation. The 13 bp spacing was chosen to be within the average loop translocation distance of EcoP15I (Fig. 1). The grey box indicates the helicase binding site. EcoP15I or LlaGI were pre-incubated with the DNA and then the reaction started with ATP and free biotin. If motor activity displaced the streptavidin from the biotin-labelled DNA end, the released streptavidin would be bound by the free biotin and unable to rebind the DNA. **b**, Example agarose gel (from 3 repeats) of reactions with 8 nM DNA and the order of addition shown for 855 nM streptavidin (yellow), 17.1 µM free biotin (blue), EcoP15I (from left to right, 264, 528 or 792 nM), nuclease mutant LlaGI D78A (50 nM) and/or ATP and biotin mix (4 mM and 17.1 µM, respectively). DNA with none, one or two streptavidin molecules can be separated. The average relative intensities of the three species were quantified (*n* = 3, errors, s.d.). Free biotin was sufficient to prevent streptavidin binding to the DNA ends (lane 3). Increasing concentrations of EcoP15I did not displace the streptavidin (lanes 4-6). Contrastingly, LlaGI can displace >80% of the streptavidin from one DNA end (lane 7) consistent with the expected directional translocation. Lane 2 shows incomplete biotin labelling of both DNA ends. Lanes 1 and 8 are the same control marker.

To evaluate the downstream DNA, we measured the cleavage of linear substrates that had varying downstream lengths between an EcoP15I site and a capped end and also had an upstream site for the related Type III enzyme EcoPI producing a tail-to-tail inverted repeat (Fig. 3c). This substrate could only be cut at the EcoPI site if both enzymes were added and both DNA ends were capped with streptavidin to prevent dissociation during sliding (Fig. 3d). EcoPI site cleavage was only observed with ≥21 bp of downstream DNA and was maximally activated with ≥25 bp (Fig. 3e). EcoP15I site cleavage cannot occur because either there is insufficient downstream DNA to accommodate the sliding EcoPI or the cleaved DNA cannot be resolved from the uncut substrate. End-capping did not block DNA binding (Supplementary Fig. 12) and EcoP15I could not displace streptavidin as seen with other helicases (Extended Data Fig. 4)^45^, indicating that a lack of DNA cleavage was not due to weaker site binding or the loss of end capping.

**Extended Data Fig. 5:**
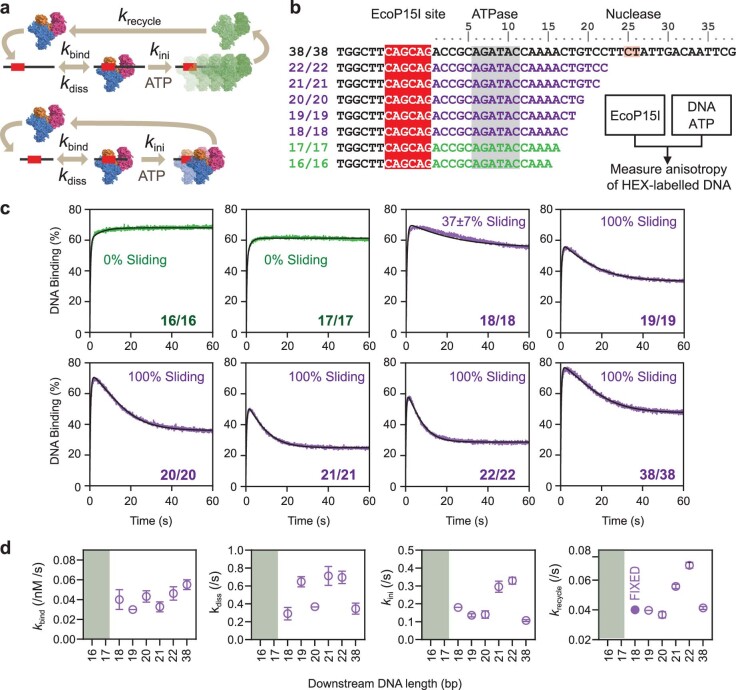
Downstream DNA-length dependence of sliding state formation. **a**, EcoP15I-DNA binding and ATP-dependent dissociation schemes that either do (*upper*) or do not (*lower*) produce a sliding state^30^. **b**, Substrates with varying downstream DNA lengths shown as the duplex MS sequence. Substrates named according to Supplementary Fig. 13. Cartoon shows the stopped-flow rapid mixing regime. **c**, Binding kinetics of EcoP15I to its target site with ATP. Data is the average of three independent repeats. The 16/16 and 17/17 data were fitted to a double exponential by nonlinear regression: 16/16 Plateau = 68.1 ± 0.0 %, Fast phase = 88.0 ± 0.3 %, *k*_fast_ = 2.90 ± 0.03 s^–^^1^, *k*_slow_ = 0.20 ± 0.01 s^–^^1^; 17/17 Plateau = 61.3 ± 0.1 %, Fast phase = 75.1 ± 1.2 %, *k*_fast_ = 3.50 ± 0.08 s, *k*_slow_ = 0.64 ± 0.10 s^–^^1^ (errors, s.e.m.). The 18/18 to 38/38 data were fitted to the *upper* scheme in panel a by numerical integration (parameters in panel d). For 18/18, only a fraction of dissociated species underwent the recycling. **d**, Fitted mean parameter values for the 18/18 to 38/38 data from panel c (errors, s.d., *n* = 3). For fitting the 18/18 data, *k*_recycle_ was fixed at 0.04 /s and a fraction of dissociated species could bypass this state (37 ± 7%, errors, s.d., *n* = 3).

We next exploited the observation that sliding off a DNA end produces a free conformation that must slowly isomerize before it can rebind DNA (Extended Data Fig. 1d)^30^. Using stopped-flow fluorescence spectroscopy to measure binding with ATP to an oligoduplex with 38 bp downstream (38/38, named according to MS/TS downstream lengths; Supplementary Fig. 13), the formation of this ‘sliding conformation’ could be detected as a transient maximum followed by a lower steady state (Extended Data Fig. 5a,b)^30^. Similar transients were observed using 19/19 to 38/38 spacings and could be fitted to the previously determined kinetic model^30^, consistent with ∼100% formation of a sliding conformation (Extended Data Fig. 5c,d). At 18 bp, the data could be approximated by a fit where ∼37% of events produced sliding. At 16–17 bp, the absence of transients indicated that a sliding conformation was not produced and the data could be fitted to an exponential consistent with a two-state model (free and bound). The difference between the threshold at 18–19 bp and at 21–25 bp in Fig. 3e could be because either streptavidin inhibits sliding state formation between 18–24 bp or the ‘conformationally locked’ sliding state can form but requires additional downstream DNA (≥21 bp) to properly load onto the DNA and allow long-range 1D diffusion. In either case, the data were consistent with additional downstream DNA translocation after loop collapse being required to establish sliding.

**Fig. 4:**
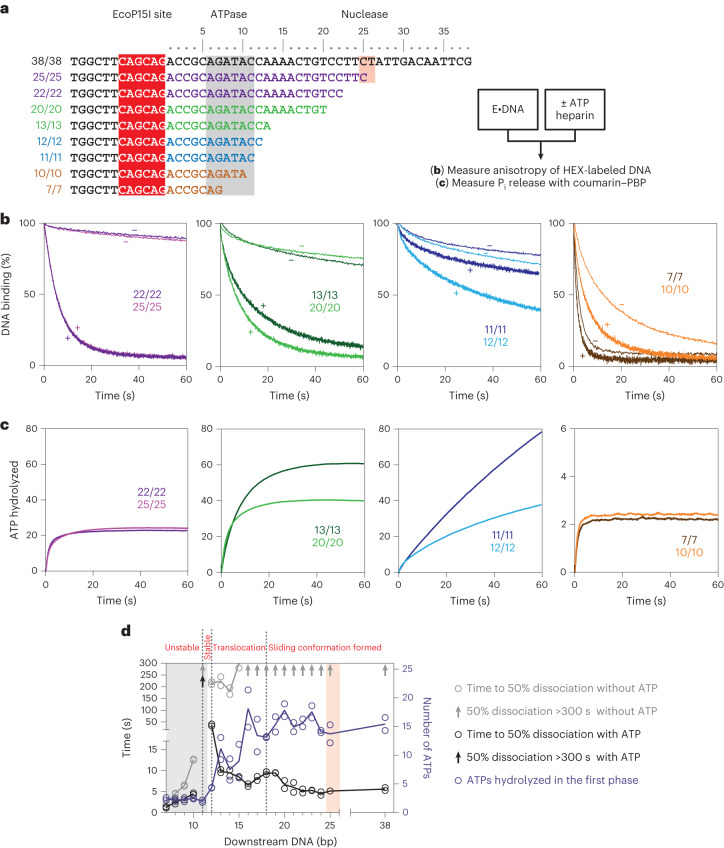
Effect of downstream DNA on DNA binding and ATPase activity. **a**, Example substrates with varying downstream DNA lengths shown as the duplex MS sequence with colors indicating substrate classifications. Data are grouped into columns according to classifications (see main text). Substrates are named according to Supplementary Fig. 13. P_i_, phosphate. **b**, Examples of the release of prebound enzyme from its target site with heparin trap and with (+) or without (−) ATP, measured using HEX anisotropy. Data are the average of two independent repeats (Supplementary Fig. 8). **c**, Examples of ATP hydrolysis by prebound enzyme measured by phosphate release. Data were corrected for the heparin background (Supplementary Fig. 9) apart from 11/11 and 12/12, which are shown uncorrected. **d**, Downstream length-dependence of DNA dissociation measured as the time to reach 50% with or without ATP and the number of ATPs consumed during the first ATPase phase. Circles show fitted parameters from two repeat experiments. Full kinetic parameters are in Supplementary Fig. 14.

To further explore this, we measured DNA dissociation and ATPase activity^30,31^ using oligoduplexes with 7–38 bp of downstream DNA (Fig. 4a). Reactions were initiated on prebound enzyme–DNA complexes by ATP addition with heparin to sequester free/dissociated enzyme. We split the results into four groups with similar kinetic characteristics. Representative data are shown in Fig. 4, full datasets are shown in Supplementary Figs. 8 and 9, and data fitting constants are shown in Fig. 4d and Supplementary Fig. 14.

#### Substrates 22/22 to 38/38

These downstream lengths matched those that produced 1D communication (Fig. 3e). All substrates showed the previously observed characteristics^30,31^ of stable protein-DNA binding without ATP and rapid dissociation with half-lives of <6 s with ATP (Fig. 4b, column 1; Fig. 4d). Biphasic ATPase activity was observed, with a rapid first phase resulting in hydrolysis of ∼15 ATPs followed by a slower phase that matched the dissociation kinetics and consumed ∼10 ATPs (Fig. 4c, column 1; Supplementary Fig. 9). The slower phase most likely reflects protein interactions with DNA ends (discussed further in ref. ^31^).

#### Substrates 13/13 to 21/21

These downstream lengths produced stable protein complexes with dissociation accelerated by ATP but at reduced rates (Fig. 4b, column 2; Fig. 4d). The first-phase ATPase kinetics were largely unchanged, but the second-phase ATP hydrolysis rate increased, matching the slower dissociation kinetics so that more ATP was consumed before dissociation (Fig. 4c, column 2; Fig. 4d and Supplementary Fig. 9). This longer-lived second phase may reflect the failure to properly initiate 1D diffusion (Fig. 3e).

#### Substrates 11/11 to 12/12

These substrates provided sufficient DNA for complete initial helicase contacts (Fig. 3a) but probably could not support loop translocation because dissociation was barely stimulated by ATP (Fig. 4b, column 3). The stabilized DNA-bound state produced elevated ATP hydrolysis, particularly for substrate 12/12 (Fig. 4c, column 3). DNA contacts, possibly with a DNA end, support an uncoupled ATPase activity that eventually leads to dissociation without remodeling.

#### Substrates 7/7 to 10/10

**Extended Data Fig. 6:**
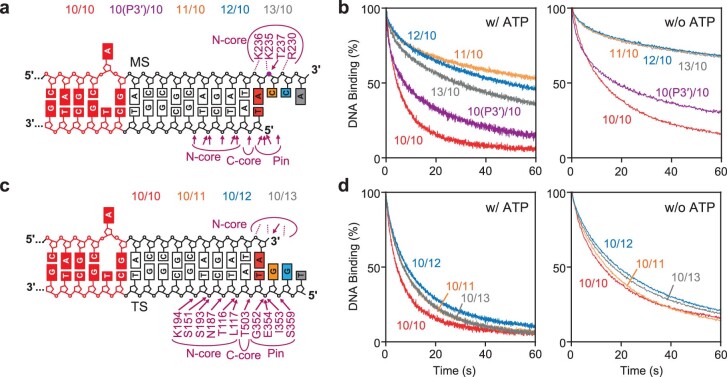
N-core RecA contacts to the methylating strand backbone are necessary for stable DNA association. **a**, Cartoon of the sequences of the DNA substrates with an extended MS (upstream DNA sequence not shown). 10(P3ʹ)/10 had an MS with 10 nt downstream and a 3ʹ phosphate. Curved lines show regions of Res-DNA contact from PDB:4ZCF^15^ with arrows indicating side chain contacts and dotted lines indicating main chain contacts. **b**, Release of prebound enzyme from its target site with heparin trap and with (*left*) or without (*right*) ATP measured using HEX anisotropy. The weakly-bound 10/10 DNA was extended on the MS strand. An additional 3ʹ phosphate produces a small increase in stability but the additional base at position 11 is also important, possibly to help orient the phosphate-T237 interaction. Further extension of the MS has only a moderate effect. **c**, Cartoon of DNA substrates with an extended TS (upstream DNA sequence not shown). **d**, Release of prebound enzyme from its target site with heparin trap and with (*left*) or without (*right*) ATP measured using HEX anisotropy. Extension of the TS strand had little effect compared to the weakly-bound 10/10 DNA. One representative dataset shown from two independent repeats.

These substrates provided partial helicase–DNA contacts (Fig. 3a), and binding was markedly weakened even in the absence of ATP (Fig. 4b, column 4). Very little ATPase activity could be measured before dissociation occurred (Fig. 4c, column 4). These data suggest that helicase–DNA contacts are vital for stable DNA–holocomplex association. By extending either the MS or the TS on the weakly bound 10/10 substrate, we identified that phosphate 11 on the MS that contacts Res T237 from the RecA N-core is critical for stable binding (Extended Data Fig. 6). The moderate acceleration of dissociation by adding ATP (Fig. 4d) may be due to a nonhelicase effect of the nucleotide, for example, by binding in the AdoMet binding pocket of the Mod subunits.

In summary (Fig. 4d), ATP hydrolysis drives DNA dissociation consistent with the release of Mod–DNA contacts. The first phase ATP burst reaches a maximum when there are ∼5 bp downstream of the leading edge of the helicase (that is, 17 bp downstream of the EcoP15I site), which also provides the biggest difference between dissociation rates with or without ATP. However, additional downstream DNA contacts and/or translocation are needed to form a sliding conformation (+3 bp; Extended Data Fig. 5) or to support 1D communication and the most rapid DNA dissociation (+5 bp; Figs. 3e and 4d).

### Both DNA strands are required for DNA translocation

**Fig. 5:**
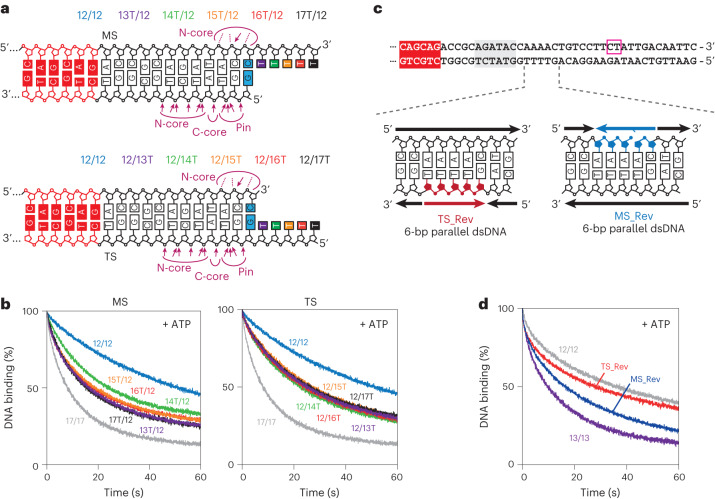
DNA strand dependence of translocation. **a**, Oligoduplex substrates (upstream 6 bp of DNA not shown) with a poly(dT) extended MS (top) or TS (bottom). Curved lines show regions of Res–DNA contact from PDB 4ZCF (ref. ^15^) with arrows indicating side chain contacts and dashed lines indicating main chain contacts. **b**, Release of prebound EcoP15I from its target site with ATP and the heparin trap measured using HEX anisotropy. The 12/12 DNA was extended on either MS (left) or TS (right) and compared to 12/12 and 17/17. **c**, Cartoon of 50-bp oligoduplex substrates (upstream 6 bp of DNA not shown) with a 5-nucleotide stretch of reversed-polarity backbone on the MS (MS_Rev) or TS (TS_Rev) immediately downstream of the initial helicase-like motor-binding site (Fig. 3a). **d**, Release of prebound enzyme from its target site with ATP and the heparin trap measured using HEX anisotropy. The reversed-polarity DNAs were compared to 12/12 and 13/13.

To test the roles for the TS and the MS in translocation, we followed DNA dissociation on oligoduplexes based on 12/12, which produces stable binding but not rapid dissociation, by extending either the MS or the TS by 1–5 dT residues (Fig. 5a,b). The addition of a single dT on either strand was sufficient to accelerate ATP-dependent dissociation, and the addition of further dT residues had only a small effect and activity was not as efficient as on 17/17. This is consistent with the helicase-like motor requiring dsDNA for efficient translocation as we found for the related Type I RM enzymes^46^. We also tested 38/38 oligoduplexes with 5-bp runs of reversed backbone polarity on either the TS or the MS, 2–6 bp downstream of the initial helicase contacts (Fig. 5c,d). TS modification resulted in slower ATP-dependent dissociation, similar in rate to that of the hyperstable 12/12 DNA-complex. This is consistent with a principal role for the TS in directional motion. MS modification had a smaller inhibitory effect. Disruption of the dsDNA structure by the patch of parallel DNA might affect the auxiliary role for the MS in motor activity.

### The nuclease domain has a putative clamp-like structure

**Extended Data Fig. 7:**
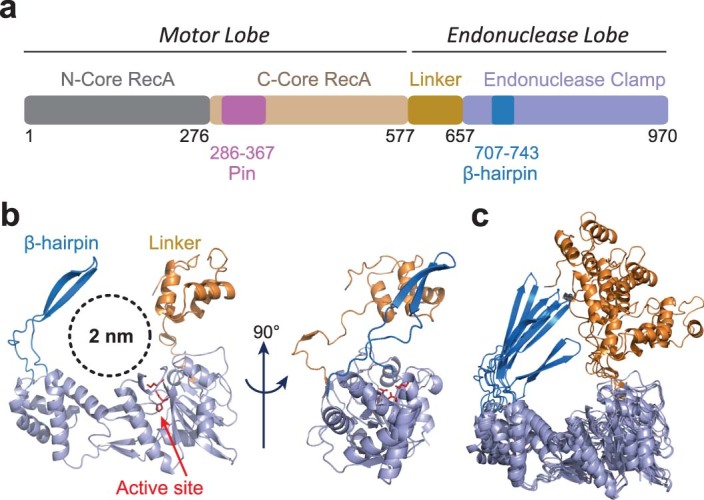
Structural models for the nuclease lobe of the EcoP15I Res subunit. **a**, Domain arrangement of Res determined from the crystal structure^15^ and the Alphafold 2 structural predictions (Supplementary Figs. 15, 16)^47,48^. **b**, Ribbon diagram views of an Alphafold 2 prediction of the Endonuclease Lobe structure shown in two orientations. The dashed circle indicates the diameter of B-form dsDNA that could fit within the clamp formed by the β-hairpin and Linker, placing the nucleic acid near the PDExK nuclease active site residues P892, D898, E916 and K918 (red sticks). **c**, Five predicted models (Supplementary Fig. 16) aligned using the nuclease fold showing relative disorder/lower confidence in the placement of the β-hairpin and Linker.

**Extended Data Fig. 8:**
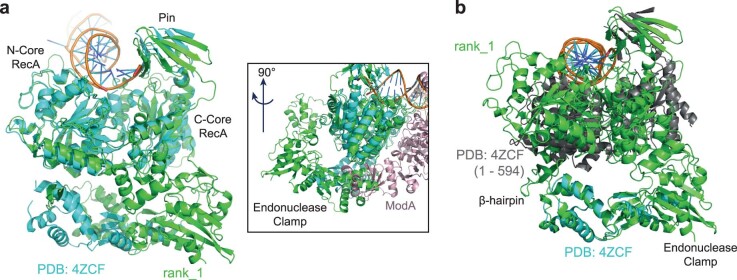
Alignment of the rank_1 AlphaFold 2 structure to the EcoP15I crystal structure. **a**, Alignment of rank_1 (green) to PDB:4ZCF^15^ (blue) via the helicase lobe (residues 1–594). (*inset*) Rotated alignment to show that the Endonuclease Lobe of rank_1 clashes with ModA of PDB:4ZCF (pink). Clashes are also seen for other predicted structures. **b**, Alignment of rank_1 (green) to the partial nuclease regions of PDB:4ZCF^15^; (discontinuous residues 620–633, 667–705, 744–774, 804–810) in blue with the helicase lobe (residues 1–594) in grey.

The importance of downstream DNA in establishing diffusive states (Fig. 3 and Extended Data Fig. 5) suggests a role for the nuclease domain in long-range communication. To provide further insight, we used AlphaFold2 Colab with MMseqs2 (refs. ^47,48^) to predict the Res structure and reveal the C-terminal nuclease lobe that was not resolved in the crystal structure (Extended Data Fig. 7 and Supplementary Figs. 15 and 16). In five ranked predictions, the helicase domain closely aligned to the crystal structure, while the nuclease lobe could be aligned with the partial elements from the crystal structure (Extended Data Fig. 8). The nuclease lobe was located in different positions relative to the motor lobe in the predictions, possibly consistent with flexibility of the nuclease that led to its absence from the solved crystal (Supplementary Fig. 15), and contain several distinct features common to all models (Extended Data Fig. 7): (1) a C-terminal 80-residue linker comprising a central ⍺-helical bundle surrounded by disordered loops (Supplementary Fig. 16); (2) a nuclease subdomain with the characteristic αββα fold and PDExK active site; (3) an ⍺-helical bundle that extends from the center of the nuclease subdomain; and (4) a β-hairpin that extends from the ⍺-helical bundle. The four subdomains form a clamp-like arrangement with jaw dimensions that could accommodate dsDNA and place it close to the nuclease active site with the linker and β-hairpin encircling the DNA (Extended Data Fig. 7b). When comparing the predicted models aligned at the nuclease subdomain, the linker and β-hairpin showed a high degree of flexibility and possible disorder (Extended Data Fig. 7c and Supplementary Fig. 16) that would suggest that additional stabilization of the jaw would be required for the formation of a long-lived sliding clamp.

## Discussion

Using ultrafast single-molecule twist measurements based on plasmonic DNA origami nano-rotors combined with ensemble assays, we demonstrated that the SF2 helicase-like ATPase of the Type III RM enzyme EcoP15I uses short-range (5–22 bp) dsDNA loop translocation to remodel an MTase–DNA complex. Uniquely for a remodeling enzyme, this activity establishes a long-range DNA diffusion conformation. Our data integrate previous alternative Type III RM translocation-based models within the diffusion model and rule out a simple conformational change in the absence of translocation (‘molecular switch’). Motion of the Res subunit was found to be downstream of the Mod–DNA complex, in agreement with 3ʹ–5ʹ TS tracking^15^. Consistent with other SF2 helicases, the 3ʹ–5ʹ translocating strand is the principal motor contact, but dsDNA appears necessary for translocation (Fig. 5). We suggest that, similar to the SF2 helicase-like motor of Type I RM enzymes^46^, contacts to the 5ʹ–3ʹ strand by the Res motor are important for processivity (most likely via N-core RecA contacts^15^).

**Fig. 6:**
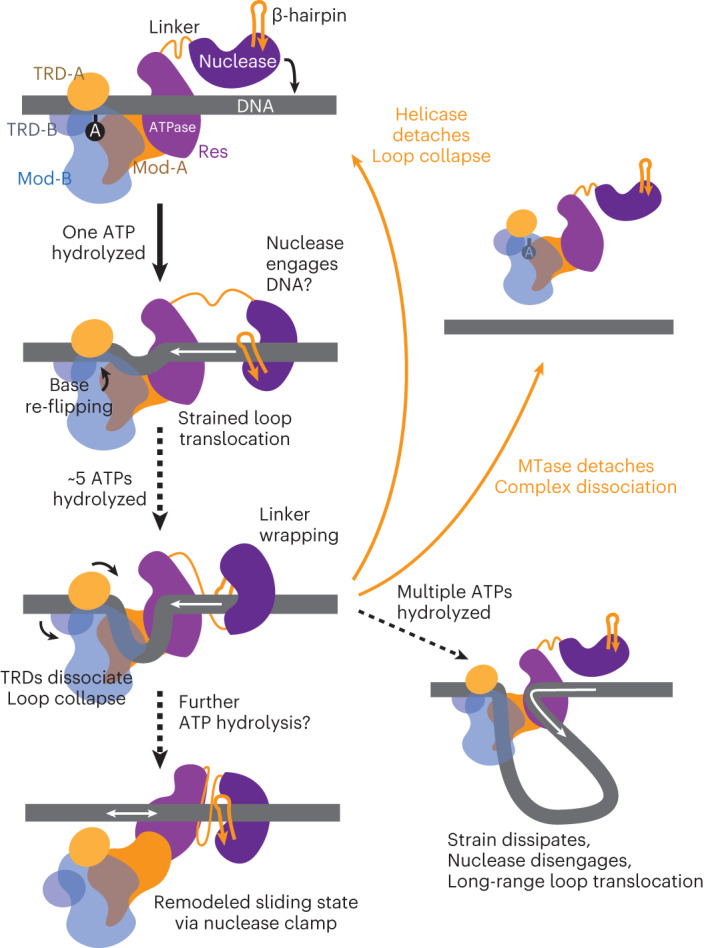
Model for activation of DNA sliding by EcoP15I by short-range dsDNA loop translocation. Given the well-defined *cis* cleavage site 25–26 bp downstream of the recognition site on the MS^50^, the nuclease domain should engage the DNA early in the pathway. Hydrolysis of the first ATP and the start of loop translocation returns the flipped adenine to the DNA. Further translocation leads to strain, resulting in helicase dissociation and loop collapse; dissociation of the whole complex; dissipation of strain and formation of a larger translocating loop; or dissociation of the TRDs, loop collapse and translocation without looping. Only the latter allows the motor domain to engage the nuclease to remodel the DNA contacts and to produce a diffusing state that can activate cleavage at a distant DNA site. During translocation following the helical path, the linker may be wrapped around the DNA to support sliding clamp formation, as seen for Mfd^7^.

Overall, our data support two-step formation of the diffusing state (Fig. 6): initial release of the Mod subunits from the target site (remodeling of Mod-DNA contacts) followed by the establishment of an enzyme conformation that supports long-lived diffusion. For the first step, we demonstrated that the first ATP hydrolyzed produced an environmental change in the flipped target adenine (Fig. 2d), possibly returning the base to the helix as a primary remodeling step. Further ATP hydrolysis then produced downstream DNA loop translocation that remodels the TRD, leading to release of the DNA loop (Fig. 2c). The MTase–DNA contacts alone did not support stable DNA binding. Rather, both the Mod–DNA and helicase–DNA contacts were necessary to stabilize the initial nucleoprotein complex (Fig. 4), in particular, MS backbone contacts by N-core RecA (Extended Data Fig. 6). If MTase–DNA binding is relatively weak, the predominant loop dissociation after 5 bp of translocation is often due to release of the MTase from the site. However, observed repetitive loop translocation events might indicate the possibility for dissociation of the motor before TRD remodeling followed by immediate re-initiation.

For the second step—establishing the diffusion state—it is important to note that ATPase-driven MTase release of the site required only ∼5 bp of dsDNA translocation (the predominant maximum translocation distance in the single molecule experiments; Fig. 1) while long-range communication relied on >8 bp downstream of the helicase (Fig. 3e and Extended Data Fig. 5). The saturated first ATPase burst of ∼15 ATPs measured here (Fig. 4d) and a typical helicase-like motor coupling of 1 ATP per base pair would correspond to ∼15 bp of translocation. If further motor activity after TRD release is required to establish sliding, it cannot be in the form of loops. Interestingly, the 15-bp translocation corresponds to the cleavage loci, and thus the putative binding site of the nuclease lobe (Extended Data Fig. 7). Given the well-defined cleavage at 25–26 bp downstream of the recognition site, we expect that the nuclease lobe engages the DNA early in the pathway (Fig. 6). The motor domain would thus ‘bump into’ the nuclease, potentially remodeling its flexible jaw to form a stable clamp to transition into sliding. Furthermore, because we did not see negative angular displacement of the nano-rotor, we speculate that translocation along the DNA helix towards the nuclease will also wrap the disordered ‘linker’ around the DNA, providing additional stabilization during motion as seen previously for MfD^7^. The low-frequency larger loop states (>40 bp) may occur when TRD-A does not release the site and the nuclease lobe either has not engaged the DNA or is displaced (Fig. 6). These rare events could explain the loop translocation species observed by fast scanning AFM^42^. It is not clear whether such long-range loop translocation states could activate cleavage at a distant site.

Altogether, our model suggests that the Type III motor has dual remodeling functions (Fig. 6). First, it remodels the MTase to release it from the recognition site, and second, it remodels the nuclease lobe to release it from the cleavage site, accompanied by sliding clamp formation. Long-lived 1D diffusion would then be facilitated by the nuclease lobe and/or linker encircling the DNA. Hydrolysis of the first ATP could trigger cleavage site binding by the nuclease lobe. This is consistent with observations that a site-bound enzyme needs to be able to hydrolyze ATP for cleavage to be activated following collision with a sliding enzyme^37,44^. To reveal the protein conformations necessary for long-lived DNA sliding and cleavage, future experiments should focus on the structural outcome of remodeling and collision.

## Methods

### Proteins

Wild-type EcoP15I and EcoPI were purified as described previously^37,51^. Labeling of K339 of EcoP15I Mod was by orthogonal amino acid labeling followed by click chemistry^52^. The K339 codon of *ecoP15Ires* was mutated in pSP11.3 to an amber stop codon by QuikChange mutagenesis (Agilent Technologies) using primers 5ʹ-CATCCAAAAACTTAGAAGCCATGCAAACAACC-3ʹ and 5ʹ-GGTTGTTTGCATGGCTTCTAAGTTTTTGGATG-3ʹ and confirmed by sequencing of the full operon. *E. coli* BL21(DE3) cells were co-transformed with pSP11.3 339TAG and pCDF PylST^53^ and single colonies were selected on LB agar with 50 µg ml^-1^ spectinomycin and 50 µg ml^-1^ ampicillin. *N*_6_-[(2-azidoethoxy)carbonyl]-l-lysine (N3-Lys, 0.1 M; SiChem) was prepared in 0.1 M NaOH and filter-sterilized. Four 2.5-liter flasks of 500 ml LB with 50 µg ml^-1^ spectinomycin, 50 µg ml^−1^ ampicillin and 1 mM N3-Lys were inoculated with one colony each and grown for 22 h at 37 °C while shaking at 250 r.p.m. The overnight cultures were pelleted at 4,410*g* for 30 min at 4 °C, washed with 25 ml of 10 mM Tris-Cl (pH 8.0), 100 mM NaCl and 1 mM ethylenediaminetetraacetic acid (EDTA) and re-pelleted into a 50-ml sterile Falcon tube at 4,500*g* for 20 min at 4 °C.

The cell pellet was resuspended in 100 ml of 100 mM Tris-Cl (pH 8.0), 150 mM NaCl, 5 mM MgCl_2_, 2.5 mM EDTA, 3 mM dithiothreitol (DTT), 9 µg ml^−1^ phenylmethysulfonyl fluoride and EDTA-free protease inhibitor tablets according to the manufacturer’s instructions (Roche). Cells were lysed in 50-ml batches using a Sonics Vibra-Cell sonicator fitted with a 0.5-inch tip at 60% amplitude, with 10 s on and 10 s off for 2 min. Soluble and insoluble fractions were separated using the Optima L-80 XP ultracentrifuge (Beckman Coulter) fitted with the Ti70 rotor, prechilled to 4 °C, at 112,000*g* for 110 min. The supernatant was dialyzed against 2 liters of buffer B1 (10 mM Tris-Cl (pH 8.0), 50 mM NaCl, 0.1 mM EDTA, 1 mM DTT) using 10-kDa molecular weight cut-off (MWCO) SnakeSkin dialysis tubing (Pierce) for 120 min at 4 °C. The dialyzed soluble fraction was loaded onto a 210-ml DEAE Sepharose-FF column (Cytivia) equilibrated with buffer B1. Proteins were eluted using a linear gradient from 50 mM to 550 mM NaCl at 5 ml min^−1^. Fractions containing EcoP15I Res and Mod were chosen by SDS–PAGE and dialyzed against 2 liters of buffer C1 (10 mM Tris-Cl (pH 8.0), 50 mM NaCl, 1 mM DTT) for >2 h at 4 °C. Dialyzed supernatant was loaded onto a 20-ml HiPrep Heparin FF 16/10 column equilibrated with buffer C1. EcoP15I was eluted using a linear gradient of 50 mM to 650 mM NaCl. Fractions were chosen by SDS–PAGE concentrated to <0.5 ml and exchanged into a storage buffer (10 mM Tris-Cl (pH 8.0), 100 mM NaCl, 1 mM DTT, 10% (v/v) glycerol) using a 15-ml 50-kDa MWCO Amicon ultrafiltration device at 4,000*g*.

EcoP15I 339Lys-N3 (0.5 mg) in 223 µl storage buffer and 11.8 µl of 7 mM dibenzocyclooctyne-Cy3 (DBCO–Cy3; Sigma-Aldrich) in dimethylformamide were mixed and incubated at 25 °C in the dark for 2.5 h with gentle mixing every 30 min. The sample volume was increased to ∼1 ml with storage buffer and particulates were removed by centrifugation at 16,100*g* for 2 min at 4 °C. The labeled protein was loaded onto a 120-ml HiLoad 16/60 Superdex 200 gel filtration column (GE Healthcare) equilibrated with storage buffer and protein was eluted at 1 ml min^−1^. Fractions were chosen by SDS–PAGE and concentrated using a 15-ml 50-kDa MWCO Amicon ultrafiltration device at 4,000*g*. Using an extinction coefficient of 151,000 cm^−1^ M^−1^, the degree of labeling of the Mod_2_Res_1_ complex was calculated as 1.51 (∼75% labeling of each Mod). DNA cleavage activity was monitored using 2 nM pKA16.10 (ref. ^22^), 50 nM EcoP15I and 4 mM ATP in buffer R+ (50 mM Tris-Cl (pH 8.0), 50 mM KCl, 10 mM MgCl_2_, 1 mM DTT, 100 µg ml^-1^ BSA). Reactions were quenched at the time points indicated with 0.5 volumes of STEB (0.1 M Tris-Cl (pH 7.5), 0.2 M EDTA, 40% (w/v) sucrose, 0.4 mg ml^−1^ bromophenol blue) and heated for 10 min at 80 °C; the DNA was separated by agarose gel electrophoresis (Supplementary Fig. 4e).

LlaGI D78A was purified and provided by R. Smith^54^. 7-Diethylamino-3-((((2-maleimidyl)ethyl)amino)carbonyl)coumarin-labeled phosphate binding protein (PBP–MDCC) was produced as previously described^55^.

### Assembly of the DNA origami nano-rotor and preparation of the magnetic tweezer experiments

The nano-rotor construct was based on ref. ^43^. In brief, five different parts were ligated as follows: (1) a ∼ 620-bp biotin-modified handle; (2) a 7.5-kb dsDNA spacer; (3) the origami rotor arm; (4) a 50-bp EcoP15I target sequence; and (5) a ∼ 620-bp digoxigenin-modified handle. For control measurements, a 41-bp sequence without the EcoP15I target site was used. The L-shaped origami rotor consisted of a 106-nm-long six-helix bundle stem and a 55-nm-long rotor arm with seven specific binding sites for subsequent AuNP binding. The sequence of the 7.5-kb dsDNA spacer was altered from ref. ^43^ to prevent EcoP15I cleavage. The oligonucleotides for the EcoP15I target sequence and the dsDNA spacer PCR reaction are given in Supplementary Table 1.

Flow cells were prepared using two 60 mm × 24 mm cover slides #1 (Menzel, ThermoScientific) that had been sequentially sonicated in ultrapure water, acetone and isopropanol for 10 min. Subsequently, the slides were rinsed with ultrapure water, sonicated for 20 min in 5 M KOH (potassium hydroxide), rinsed with ultrapure water and dried with pressurized nitrogen gas. The top slide contained two 2-mm holes. The bottom slide was spin coated using a 1% (w/v) polystyrene solution in toluene at 6,000 r.p.m. for 1 min, followed by baking at 150 °C for 1 h. Flow cell assembly was finalized on a hot plate at 120 °C, organizing the two cover slides and a Parafilm spacer (containing a cut out flow cell channel) in a sandwich. The surface was functionalized by incubation for 24 h with 50 µg ml^−1^ anti-digoxigenin (Bio-Rad) solution in PBS (137 mM NaCl, 2.7 mM KCl, 8 mM Na_2_HPO_4_ and 2 mM KH_2_PO_4_) and with a 20 mg ml^−1^ BSA solution (New England Biolabs, NEB) in NEB storage buffer (20 mM Tris-Cl (pH 8), 100 mM KCl, 0.1 mM EDTA and 50% (v/v) glycerol) for an additional 24 h. Following installation into the microscope, the flow cell was washed with 1 M NaCl and incubated with 3-µm-diameter carboxylated polystyrene microparticles (Invitrogen) in 1 M NaCl for ∼12 h to allow adsorption.

AuNP of 50 nm in diameter for use with the nano-rotor were synthesized and functionalized with 20-nucleotide 3ʹ-thiol-modified poly-thymidine oligonucleotides^56,57^. The DNA-coated AuNPs were subsequently incubated in a 5:1 molar ratio with the nano-rotor construct in nano-rotor buffer (5 mM Tris-Cl (pH 8), 11 mM MgCl_2_ and 1 mM EDTA) and then heated to 40 °C and slowly cooled to 23 °C. Magnetic beads (1.05 µm in diameter; 2 µl) (MyOne, Invitrogen) were washed twice with PBS followed by incubation with 0.2 fmol AuNP–nano-rotor construct at room temperature for 5 min in preparation buffer (10 mM Tris-Cl (pH 8), 150 mM NaCl, 6 mM MgCl_2_, 0.01% (w/v) Pluronic F-127 and 0.1% (v/v) Tween-20). The magnetic bead–nano-rotor construct was then washed with preparation buffer and resuspended in 100 µl preparation buffer.

Directly before measurement, the flow cell was sequentially flushed with preparation buffer and nano-rotor buffer, followed by addition of 50 µl of the magnetic bead–nano-rotor construct, and then the preparation was incubated for 5 min. Unbound beads were removed by flushing with 300 µl reaction buffer (50 mM Tris-Cl (pH 8), 50 mM KCl, 10 mM MgCl_2_ and 0.1 mg ml^-1^ BSA) supplemented with 8 µg ml^−1^ catalase (Sigma-Aldrich/Merck) and 20 µg ml^−1^ glucose oxidase (Sigma-Aldrich/Merck) to prevent DNA damage during laser illumination.

### Ultrafast single-molecule twist measurements

Twist measurements were performed using a home-built setup combining magnetic tweezers and scattered light microscopy (Fig. 1a)^43,58^. Force on the magnetic bead–nano-rotor construct was generated by a magnet pair (W-05-N50-G, Supermagnete) modulated using a computer-controlled stage (M-122.2DD, Physik Instrumente). DNA length was monitored in real time by simultaneously measuring the position of the magnetic bead and a surface-bound 3-µm-diameter polystyrene microparticle within a 160 ×160 pixel area at 1,000 Hz using an EoSens CL MC-1362 CMOS camera (Mikrotron) with custom-developed software (LabVIEW 2016 64-bit, National Instruments) and applying CUDA-based GPU-assisted real-time particle tracking^59^. The signal of an AuNP bound to one nano-rotor was monitored within a 32 × 32 pixel area at 3,947 Hz using an ORCA-Flash4.0 V2 sCMOS camera (Hamamatsu). To determine the rotational position of the nano-rotor at each timeframe, the AuNP intensity profile was fitted to a two-dimensional Gaussian profile within a 13 × 13 pixel area, giving a circular position distribution. The nano-rotor angle was then determined by the polar angle of the AuNP position with respect to the center of the circular position distribution.

For ultrafast twist measurements, a suitable nano-rotor was selected that had the expected apparent DNA length of ∼2.9 µm, a fluctuating AuNP and constrained rotational fluctuations. Subsequently, a force of ∼3 pN (or ∼6 pN) was applied to the nano-rotor. Control measurements without EcoP15I were conducted for at least 250 s for each investigated molecule. EcoP15I solution (100 µl; 4.66 nM) in reaction buffer without ATP was flushed into the flow cell followed by the detection of rotational fluctuations for at least 250 s. Subsequently, 100 µl of 4.66 nM EcoP15I solution in reaction buffer supplemented with 4 mM ATP was flushed into the flow cell followed by the detection of rotational fluctuations. The ‘DNA-free’ and ‘DNA-bound’ states were determined using a modified version of vbFRET (June10)^60^. The radians to base-pair conversion assumed 0.598 rad per base pair.

### DNA cleavage assays for testing long-range communication

For Fig. 3c–e, DNA substrates were generated by PCR using pairs of biotinylated and/or nonbiotinylated primers (Supplementary Table 2) and a synthetic 1,000-bp fragment with EcoP15I and EcoPI sites in a tail-to-tail repeat made by Integrated DNA Technologies (Supplementary Table 3). DNA cleavage was monitored at 25 °C using 8 nM DNA, 855 nM streptavidin, 50 nM EcoP15I, 100 nM EcoPI and 4 mM ATP in buffer R + . Reactions were started by adding streptavidin-labeled DNA to enzymes and ATP and were then quenched by adding 0.5 volumes of STEV (0.1 M Tris-Cl (pH 7.5), 0.2 M EDTA, 40% (w/v) sucrose, 0.4 mg ml^−1^ Acid Violet 7) plus 238 µM biotin. Samples were heated for 10 min at 80 °C and the DNA was separated by agarose gel electrophoresis. Bands were quantified from ethidium bromide staining using ImageQuant TL (Cytiva).

### Streptavidin displacement assays

The DNA substrate (Extended Data Fig. 4) was generated by PCR using SlideF13 and SlideRbio oligonucleotides (Supplementary Table 2) and a synthetic 1,000-bp fragment (Supplementary Table 3). Displacement activity was monitored at 25 °C using 8 nM DNA, 855 nM streptavidin, 17.1 µM free biotin, 264, 528 or 792 nM EcoP15I, 50 nM LlaGI D78A and/or 4 mM ATP in buffer R + , as indicated. Reactants were added to DNA in the order indicated and quenched at 1 h by adding 0.5 volumes of STEV. Following agarose gel electrophoresis, bands were quantified from ethidium bromide staining using ImageQuant TL (Cytiva).

### Measuring protein–DNA interactions by band-shift assays

Oligonucleotides (100 pmol) were incubated with [γ-^32^P]ATP and 10 U T4 polynucleotide kinase (PnK) for 1 h at 37 °C in T4 PnK buffer (NEB), followed by a further incubation at 80 °C for 15 min, and were then purified using Micro Bio-Spin chromatography columns (Bio-Rad Laboratories). Oligonucleotides were annealed by incubation at 95 °C for 5 min in a 1:1.1 ratio of labeled to unlabeled strands to a final concentration of 1 µM duplex DNA in TEN buffer (10 mM Tris-Cl (pH 8), 1 mM EDTA, 100 mM NaCl) followed by slowly cooling to room temperature. For testing downstream DNA (Supplementary Fig. 12a), oligonucleotide 17_Fwd (Supplementary Table 6) was ^32^P-labeled and annealed to 5ʹ-biotin-GTTTTGGTATCTGCGGTCTGCTGAAGCCA-3ʹ. For testing upstream DNA (Supplementary Fig. 12c), 5ʹ-biotin-GTGCATACTACAGCAGATACGTTCGGCCAGTACACTCACAACCTTCATACCTGG-3ʹ and 5ʹ-^32^P-CCAGGTATGAAGGTTGTGAGTGTACTGGCCGAACGTATCTGCTGTAGTATGCAC-3ʹ were annealed to make 10/38 or 5ʹ-biotin-TACAGCAGATACGTTCGGCCAGTACACTCACAACCTTCATACCTGG-3ʹ and 5ʹ-^32^P - CCAGGTATGAAGGTTGTGAGTGTACTGGCCGAACGTATCTGCTGTA-3ʹ were annealed to make 2/38.

Duplex DNA (0.1 nM) was mixed with variable concentrations of EcoP15I (0–1,000 nM) in 20 µl of reaction buffer supplemented with 100 ng µl^−1^ BSA, and with 10 nM streptavidin (100-fold excess to the biotin ends) where indicated for 20 min at 25 °C followed by the addition of 4 µl of 6⨯ loading dye (reaction buffer supplemented with 48% (w/v) sucrose and 0.036% (w/v) bromophenol blue). Reactions were loaded onto a 5% (w/v) acrylamide gel (29:1 acrylamide:bis-acrylamide) in TBM (130 mM Tris-Cl (pH 8), 45 mM boric acid and 4 mM MgCl_2_) supplemented with Rhinohide (Invitrogen) under 10 V while loading before running for 1 h at 12.5 V cm^−1^. Acrylamide gels were dried onto 3MM Chr blotting paper (Whatman) at 80 °C under a vacuum using a Model 583 Gel Dryer (Bio-Rad Laboratories) for 1 h. The dried gels were exposed in a BAS CASSETTE2 2040 (Fujifilm) and visualized using a Typhoon FLA 9500 (Cytiva). The data were fit using GraphPad Prism 9.4.0.

### Steady-state and stopped-flow FRET assays

Oligonucleotides 5ʹ-GCCAGTGAA[Cy5]TAACTGGCTTCAGCAGACCGCAGATACCAAAACTGTCCTT-3ʹ (where [Cy5] is a dT residue labeled with Cy5, provided by Eurofins) and 5ʹ- AAGGACAGTTTTGGTATCTGCGGTCTGCTGAAGCCAGTTAATTCACTGGC-3ʹ (Supplementary Table 4) were annealed. Steady-state fluorescence readings were taken using an Agilent Technologies Cary Eclipse fluorescence spectrometer with the reaction chamber at 25 °C and Cary Eclipse Scan software (v1.1(132)); excitation light slits were 5 nm and the PMT voltage was set to ‘high’. EcoP15I 339–Cy3 (75 nM) and/or 25 nM Cy5-labeled oligoduplex with 4 mM ATP as indicated were mixed in Eppendorf tubes and 120 μl was transferred to a 10-mm-pathlength quartz fluorescence cuvette for readings.

Stopped-flow experiments were performed using a TgK Scientific SF-61 DX2 Double Mixing stopped-flow system at 25 °C in single mixing mode with dual photomultiplier tubes in a T configuration with a Semrock 575/25-25 bandpass filter used to record Cy3 emission and an ET655I long-pass filter used to record Cy5 emission. The excitation wavelength (75-W mercury-xenon lamp) was set to 546 ± 3 nm for Cy3 reactions or 600 ± 3 nm when only Cy5 was used. Reactions were mixed, as indicated, with final concentrations of 25 nM oligoduplex, 75 nM EcoP15I 339–Cy3, 4 mM ATP and 2.5 µM heparin in buffer R. Data were collected using Kinetic Studio 5.1.0 (TgK Scientific), with three traces from each experiment averaged, and each experiment was repeated twice. Data were fit using GraphPad Prism 9.4.0.

### Steady-state and stopped-flow 2-aminopurine fluorescence assays

Oligonucleotides 5ʹ-TGGCTTCAGC[2-AP]GACCGCAGATACCAAAACTGTCCTTCTATTGACAATTCG-3ʹ (where [2-AP] is 2-aminopurine, provided by Integrated DNA Technologies) (Supplementary Table 4) and 38_Rev (Supplementary Table 5) were annealed. Steady-state fluorescence data were collected using the Cary Eclipse fluorescence spectrometer as above, with excitation light slits of 10 nm and the PMT voltage set to ‘high’. A baseline scan of buffer R was subtracted from the emission scan readings. EcoP15I (600 nM) and/or 500 nM 2-aminopurine-labeled oligoduplex with 4 mM ATP or ATPγS in buffer R were measured, as indicated.

Stopped-flow experiments were performed using the SF-61 DX2 system with a single photomultiplier tube in an L configuration with a 360-nm long-pass filter and an excitation wavelength of 311 ± 3 nm. Reactions were mixed, as indicated, with final concentrations of 125 nM oligoduplex, 150 nM EcoP15I, 4 mM ATP and/or 4 mM ATPγS in buffer R. Data were collected and analyzed as above.

### Stopped-flow 6-HEX anisotropy assay

Complementary oligonucleotides in Supplementary Tables 5 and 6 were annealed to produce 6-HEX-labeled oligoduplexes. To test the strand polarity of translocation, HEX-5′-TGGCTTCAGCAGACCGCAGATACC-3′–3′-AAAAC-5′–5′-TGTCCTTCTATTGACAATTCG-3′ (AdT-Bio) and 38_Rev (Supplementary Table 5) were annealed to make MS_Rev, and 38_Fwd_Hex (Supplementary Table 6) and 5′-CGAATTGTCAATAGAAGGACA-3′–3′-GTTTT-5′–5′-GGTATCTGCGGTCTGCTGAAGCCA-3′ (AdT-Bio) were annealed to make TS_Rev. Stopped-flow experiments were performed using the SF-61 DX2 system with dual photomultiplier tubes in a T configuration with UV-dichroic sheets set at 90 degrees, using 550-nm long-pass filters (Schott OG550) and an excitation wavelength of 540 ± 6 nm with a Glan Foucault calcite prism to polarize the excitation beam. The *G*-factor ratio was set approximately to unity^61^, and anisotropy (*r*) was calculated using:

1 $$r=\frac{{I}_{{VV}}/{I}_{{VH}}-1}{{I}_{{VV}}/{I}_{{VH}}+2}$$

where *I*_*VV*_ is emission intensity measured through the polarizer oriented parallel to the polarized excitation and *I*_*VH*_ is emission intensity measured through the polarizer oriented perpendicular to the polarized excitation. Reactions were mixed, as indicated, with final concentrations of 25 nM HEX-labeled oligoduplex, 50 nM or 75 nM EcoP15I (as indicated), and 4 mM ATP and 2.5 µM heparin in buffer R. Data were collected and analyzed as above and also by numerical integration using Berkeley Madonna software (v.8.3.18).

### Stopped-flow PBP assay

Complementary oligonucleotides in Supplementary Table 5 were annealed to produce oligoduplexes with varying downstream lengths. Stopped-flow experiments were performed using the SF-61 DX2 system with a single photomultiplier tube in an L configuration with 455-nm long-pass filters (Schott 455GG) and an excitation wavelength of 437 ± 1 nm. All solutions were prepared and stored in plastic, without the use of a pH meter to limit free phosphate contamination. ATP was prepared in 10 mM Tris-Cl (pH 8). Excess phosphate was removed by incubation at room temperature for 2 h with 30 mM 7-methylguanosine and 1 U ml^−1^ bacterial polynucleotide phosphorylase (Sigma-Aldrich) and purified using a 3-kDa MWCO ultrafiltration device (Amicon). Before each experiment, the SF-61 flow path was washed with 2 M HCl for 1 h to remove ATPase contamination. The apparatus was then rinsed with 10 ml water followed by 10 ml buffer R. A calibration curve was collected with the addition of free phosphate in 1 μM increments to 8 μM PBP–MDCC, to a total concentration of 6 μM phosphate (Supplementary Fig. 17). Reactions were mixed, as indicated, with final concentrations of 25 nM oligoduplex, 75 nM EcoP15I, 8 µM PBP–MDCC, 4 mM ATP and 2.5 µM heparin in buffer R. Data were collected and analyzed as above.

### Combined optical tweezers and confocal microscopy (C-Trap)

A 21.7-kb DNA fragment was produced by linearizing plasmid pUC18-48×601-197 (ref. ^30^) with EcoRI, filling in the 5ʹ overhangs with biotin-16–dUTP and dATP using the Klenow fragment (3ʹ → 5ʹ exo–), and removing a 1.3-kb terminal fragment by BsaI digestion. The single end-biotinylated 10.8-kb fragment lacking any EcoP15I sites was gel purified (QIAEX II gel extraction kit, Qiagen) without exposure to ethidium bromide or ultraviolet light. The oligonucleotides 5ʹ-CGGTACAGAGCTCCCTACAGCAGTAGATGGATTAGCTGC-3ʹ and 5ʹ-CGGTGCAGCTAATCCATCTACTGCTGTAGGGAGCTCTGT-3ʹ were annealed to produce an insert containing a single EcoP15I recognition site (underlined). The 10.8-kb fragment was ligated to this insert by incubating both at a molar ratio of 2.1:1, respectively, with T4 ligase overnight at 16 °C, before purification with AMPure XP beads (Beckman Coulter).

Experiments were performed using a LUMICKS C-Trap G2 system integrating a dual optical trap and confocal microscope and C1-type microfluidics flow cells. The imaging buffer used in flow cell channels 1, 2, 4 and 5 was 50 mM Tris-Cl (pH 7.9), 50 mM NaCl, 10 mM MgCl_2_, 0.01% (v/v) Tween-20 and 0.2 mg ml^−1^ BSA, whereas channel 3 contained this buffer but with an elevated NaCl concentration of 75 mM to dissociate enzymes bound at nonspecific sites. Streptavidin-coated polystyrene beads of 4.0–4.9 µm in diameter (LUMICKS) were trapped in two 1,064-nm lasers (30% power) and used to form DNA tethers. C-terminally Avi-tagged biotinylated EcoP15I (5 nM) was incubated with 25 nM streptavidin-conjugated 655-nm quantum dots (Qdot, Invitrogen) for 3 min at room temperature. This mixture was then diluted ten-fold with imaging buffer and added to channel 4. Qdots were excited with a 488-nm laser at 4% power and imaged with a 680/42-nm emission filter as kymographs along the tether axis using a pixel exposure time of 0.1 ms, line scan time of 28 ms and pixel size of 100 nm.

To establish the kymograph positional mean and s.d. of EcoP15I bound at the central recognition site, trajectories of nonsliding enzymes were determined using the ‘kymotracker’ widget within the LUMICKS Pylake Python package. Sliding events were then defined as those where enzyme positions were displaced from the mean by over 4 s.d. for five or more consecutive frames. Time zero was set as the frame before tether movement from the channel 3 (buffer-only) waypoint to the channel 5 (4 mM ATP) waypoint, and initiation times were measured up to the frame preceding the first sliding event of each kymograph.

### Statistical analysis

The *n* values for the number of events are stated in each figure where relevant. Each single-molecule experiment was carried out on at least three different DNA molecules.

### Reporting summary

Further information on research design is available in the Nature Portfolio Reporting Summary linked to this article.

## Online content

Any methods, additional references, Nature Portfolio reporting summaries, source data, extended data, supplementary information, acknowledgements, peer review information; details of author contributions and competing interests; and statements of data and code availability are available at 10.1038/s41589-023-01504-1.

### Supplementary information

Supplementary Information Supplementary Figs. 1–17 and Tables 1–6.

Reporting Summary

## Data Availability

Example data for the single-molecule and ensemble assays are presented within the paper. The full datasets that support the findings of this study are available at the University of Bristol data repository, data.bris, at 10.5523/bris.1evk6g7f9x5ec2viqondhumvnw (ref. ^62^).

## Code availability

vbFRET is available at https://vbfret.sourceforge.net/.

LUMICKS Pylake is available at https://github.com/lumicks/pylake (10.5281/zenodo.7945384).

AlphaFold 2 is available at https://colab.research.google.com/github/sokrypton/ColabFold/blob/main/AlphaFold2.ipynb.
