# Supplementary material for: Short-range translocation by a restriction enzyme motor triggers diffusion along DNA
Source: Nat Chem Biol. 2024 Jan 2;20(6):689–98. doi: 10.1038/s41589-023-01504-1 (PMC11142916; doi:10.1038/s41589-023-01504-1)
Supplement: Supplementary file 1 — Supplementary Figs. 1–17 and Tables 1–6. [file 41589_2023_1504_MOESM1_ESM.pdf]

nature chemical biology

Article https://doi.org/10.1038/s41589-023-01504-1

Short-range translocation by a restriction enzyme motor triggers diffusion along DNA

In the format provided by the authors and unedited

Table of Contents

Supplementary Fig. 1: Spatio-temporal resolution of the nano-rotor.
Supplementary Fig. 2: Binding and loop translocation events are not observed in the absence of an EcoP15I recognition site.
Supplementary Fig. 3: Analysis of the lifetimes of free and bound states of the EcoP15I-DNA interaction.
Supplementary Fig. 4: Characterisation of cyanine dye-labelled EcoP15I and DNA.
Supplementary Fig. 5: DNA loop translocation properties of cyanine dye-labelled EcoP15I are similar to wild type EcoP15I.
Supplementary Fig. 6: Fluorescence changes due to DNA binding and ATP-dependent dissociation of cyanine dye-labelled EcoP15I from DNA.
Supplementary Fig. 7: Fluorescence changes associated with EcoP15I association with a 2-aminopurine labelled oligoduplex.
Supplementary Fig. 8: ATP-dependent DNA dissociation of EcoP15I as a function of downstream DNA length measured using stopped flow anisotropy.
Supplementary Fig. 9: ATP hydrolysis by EcoP15I as a function of downstream DNA length measured using stopped flow fluorescence.
Supplementary Fig. 10: DNA loop translocation properties of wild type EcoP15I at 6 pN stretching force are similar to those obtained at 3 pN stretching force.
Supplementary Fig. 11: The effect of DNA stretching force on the initiation of sliding by EcoP15I.
Supplementary Fig. 12: EcoP15I binding to oligoduplexes.
Supplementary Fig. 13: Examples of DNA substrate nomenclature.
Supplementary Fig. 14: Downstream length-dependence of DNA dissociation and ATPase kinetics.
Supplementary Fig. 15: Predicted Aligned Error (PAE) of ranked AlphaFold 2 structures of EcoP15I Res.
Supplementary Fig. 16: Predicted local distance difference test (pLDDT) of ranked AlphaFold 2 structures of EcoP15I Res.
Supplementary Fig. 17: Conversion of raw fluorescence signal into phosphate released.

Supplementary Table 1: Oligodeoxyribonucleotide sequences used for EcoP15I target sequence, no-target sequence and PCR for dsDNA spacer.
Supplementary Table 2: Primer sequences used for PCR to make substrates in Fig. 3c-e.
Supplementary Table 3: Sequence of dsDNA fragment used as a PCR template to make the substrate in Fig. 3 and Extended Data Fig. 4.
Supplementary Table 4: Internally fluorescent labelled oligodeoxyribonucleotide sequences and complementary strands annealed to make substrates for stopped-flow spectroscopy.
Supplementary Table 5: Oligodeoxyribonucleotide sequences annealed to make substrates for stopped-flow spectroscopy.
Supplementary Table 6: Hexachlorofluoroscein-labelled oligodeoxyribonucleotide sequences annealed to make substrates for stopped-flow spectroscopy.

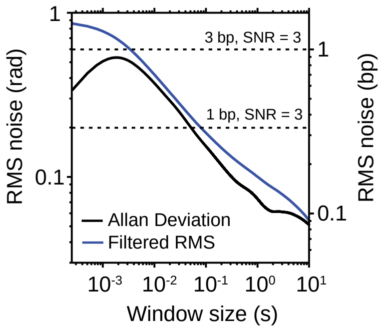

Supplementary Fig. 1: Spatio-temporal resolution of the nano-rotor. Allan Deviation (black) and root mean square (RMS) noise after filtering with a sliding average (blue). Horizontal dashed lines show the noise level at which twist changes of 3 bp or 1 bp could be resolved while assuming a signal to noise ratio (SNR) of 3. The intersections of these lines with the RMS provide information about the temporal resolution to observe these twist changes. This yields a temporal resolution of ~4 ms for 3 bp and ~80 ms for 1 bp.

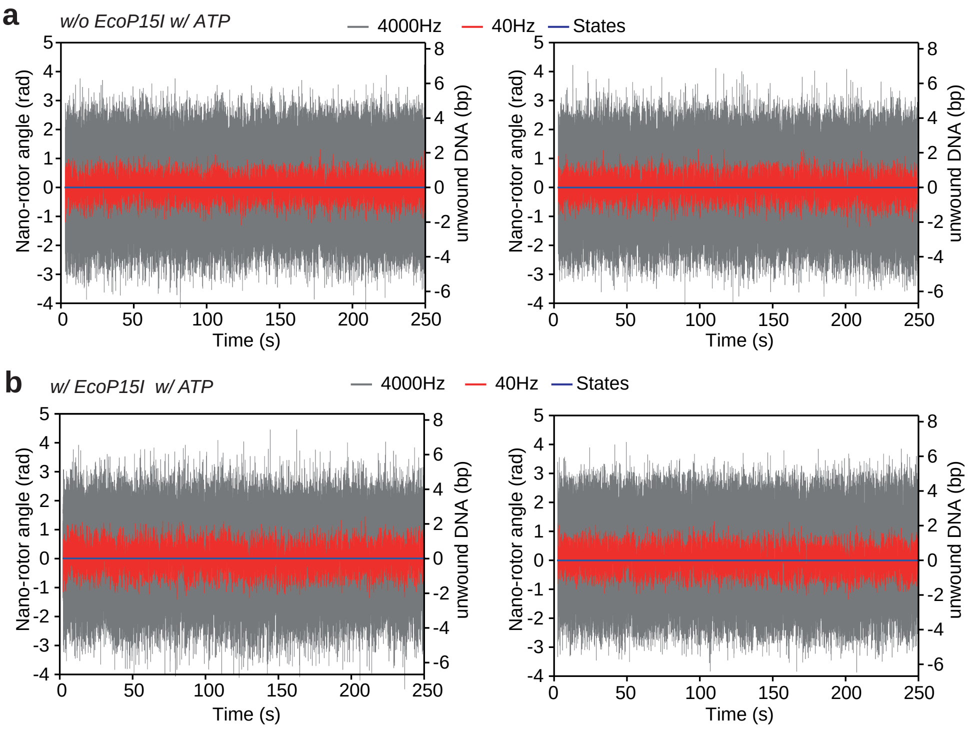

Supplementary Fig. 2: Binding and loop translocation events are not observed in the absence of an EcoP15I recognition site. a-b, Representative time trajectories with ATP (w/ ATP) of the angular position of the nano-rotor (grey, at 4000 Hz; red, after 100-point sliding average ≙ 40 Hz) without (w/o) EcoP15I (panel a) or with (/w) 4.66 nM EcoP15I (panel b). A state approximation of the trajectories was determined using vbFRET (vbFRET_June10)[61] (blue).

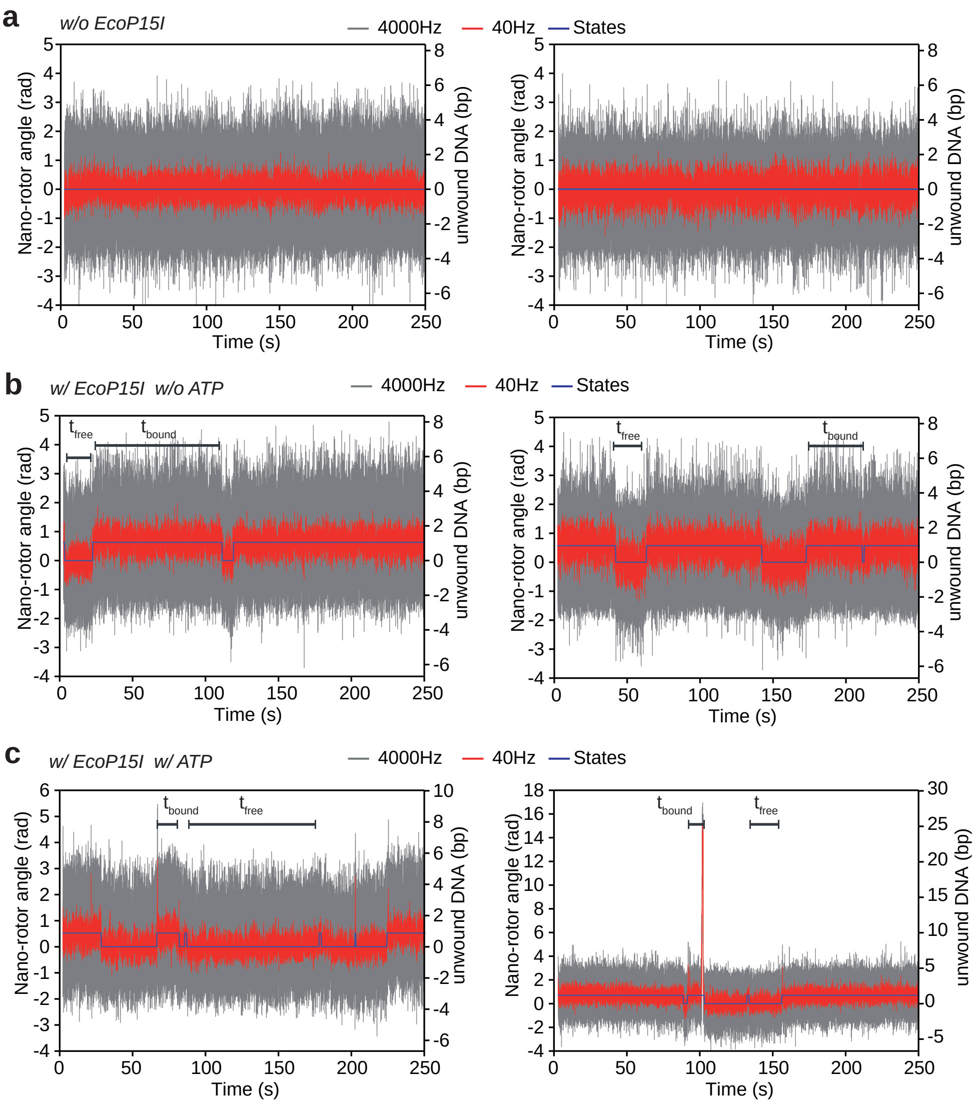

Supplementary Fig. 3: Analysis of the lifetimes of free and bound states of the EcoP15I-DNA interaction. Representative time trajectories of the angular position of the nano-rotor (grey, at 4000 Hz; red, after 100-point sliding average ≙40 Hz). A two-state approximation of the trajectories was determined using vbFRET (vbFRET_June10)[61] (blue). The analysis was done for: **a**, control measurement without EcoP15I (w/o EcoP15I); **b**, measurements with EcoP15I but without ATP (w/ EcoP15I, w/o ATP); and **c**, measurements with both EcoP15I and ATP (w/ EcoP15I, w/ ATP). Indicated are examples for the times spent in the free state (t_{free}) and the "bound" state (t_{bound}) used to determine lifetime distributions displayed in Fig. 1f.

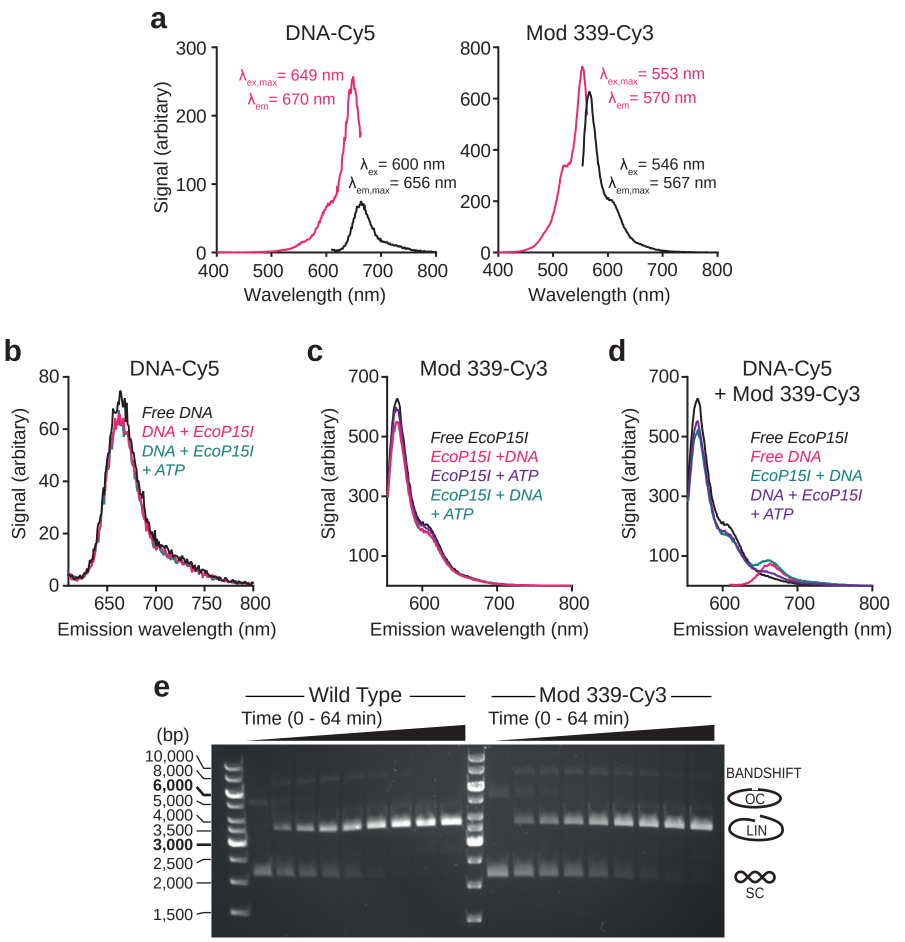

Supplementary Fig. 4: Characterisation of cyanine dye-labelled EcoP15I and DNA. a, The DNA-Cy5 substrate (Fig. 2b) was made by annealing 2AP_38_Fwd and 38_Rev (Supplementary Table 4). Steady-state fluorescence excitation (magenta) and emission (black) spectra for the Cy5 label in 25 nM DNA-Cy5 (*left*) and the Cy3 label in 75 nM EcoP15I 339-Cy3 (*right*). For excitation spectra, the wavelength was set to 570 nm for Cy3 and 670 nm for Cy5. For emission spectra, the excitation wavelength was set to 546 nm for Cy3 and 600 nm for Cy5. **b**, Steady-state fluorescence Cy5 emission spectra for 25 nM DNA-Cy5 and where indicated 75 nM unlabelled EcoP15I and 4 mM ATP. Excitation wavelength was set to 600 nm. **c**, Steady-state fluorescence Cy3 emission spectra for 75 nM EcoP15I 339-Cy3 and, where indicated, 25 nM unlabelled 38/38 oligoduplex and 4 mM ATP. Excitation wavelength was set to 546 nm. **d**, Steady-state fluorescence emission spectra for 75 nM EcoP15I 339-Cy3 and DNA-Cy5 mixed with 4 mM ATP, as indicated. Excitation wavelength was set to 546 nm except for the free DNA trace which was set to 600 nm. **e**, Cleavage activity assays for 50 nM Wild Type EcoP15I (*left*) or EcoP15I 339-Cy3 (*right*) and 2 nM pKA16. Time points were separated on a 1% (w/v) agarose gel (representative gel of 2 repeats). Time points were 0, 0.5, 1, 2, 4, 8, 16, 32 and 64 min. As well as the supercoiled (SC) substrate, nicked open circle (OC) intermediate and linear (LIN) product, a band corresponding to a DNA-protein band-shift was also observed.

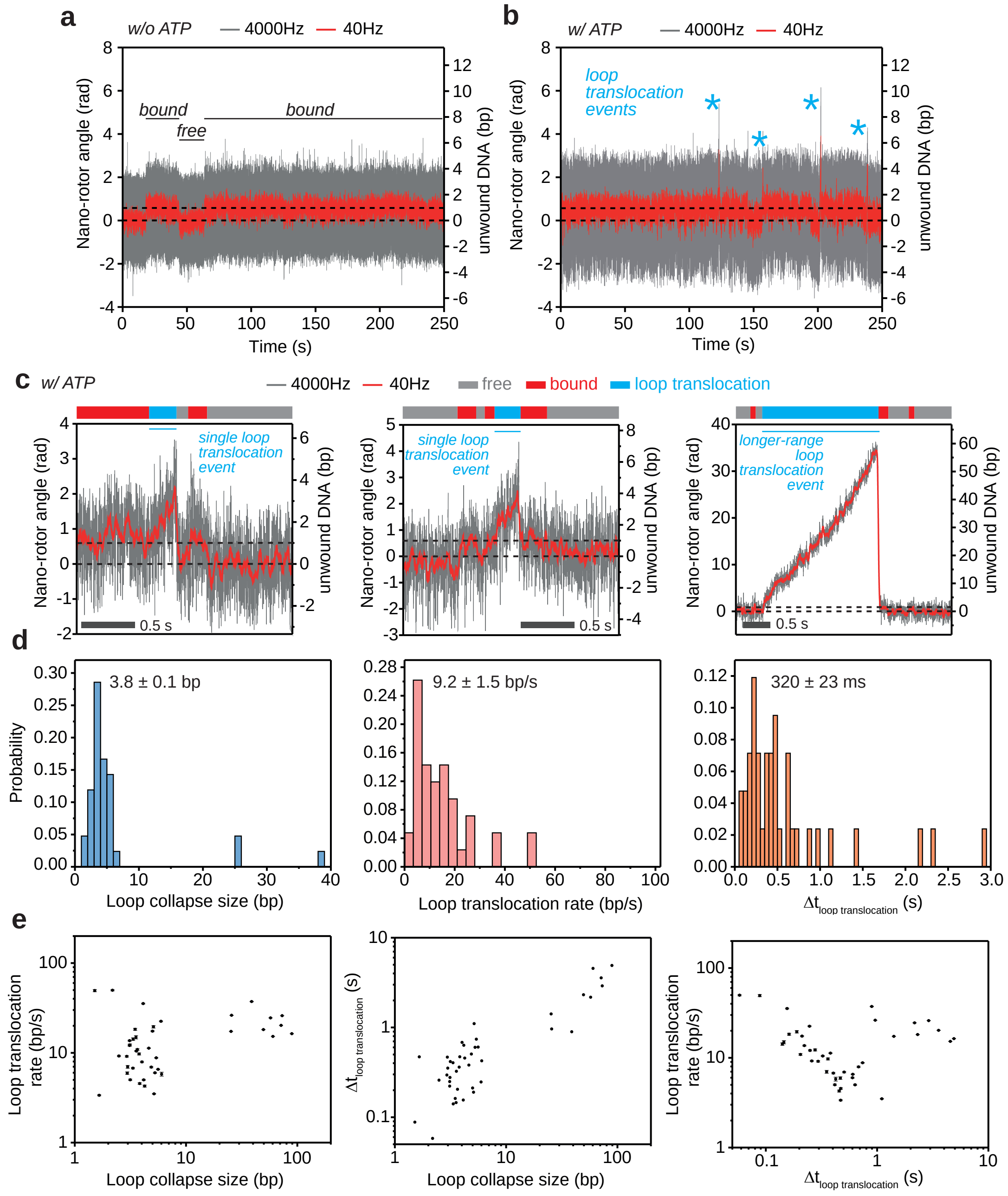

Supplementary Fig. 5: DNA loop translocation properties of cyanine dye-labelled EcoP15I are similar to wild type EcoP15I. a, Representative time trajectory of the angular position of the nano-rotor (grey, at 4000 Hz; red, after 100-point sliding average ≙ 40 Hz) with 4.66 nM EcoP15I 339-Cy3 without ATP (w/o ATP). Reversible positive rotational shifts of 0.7 ± 0.07 rad (1 ± 0.1 bp) were observed (the EcoP15I 339-Cy3 "DNA-bound" state). **b**, With ATP (w/ ATP), sawtooth-like loop translocation events were detected (blue asterisk). **c**, Representative examples of different loop translocation events including color coded identification for different EcoP15I 339-Cy3-DNA interaction states (grey, free state; red, bound state; blue, loop translocation). **d**, Maximum loop size, loop translocation rate and loop translocation time ($\Delta t_{\text{loop translocation}}$), with mean values of: 3.8 ± 0.1 bp, 9.2 ± 1.5 bp/s and 320 ± 23 ms, respectively (N = 42, error S.E.). **e-g**, Plots of the loop translocation rate vs. loop size (panel e), $\Delta t_{\text{loop translocation}}$ vs. loop size (panel f) and the loop translocation rate vs. $\Delta t_{\text{loop translocation}}$ (panel g) plotted for individual events.

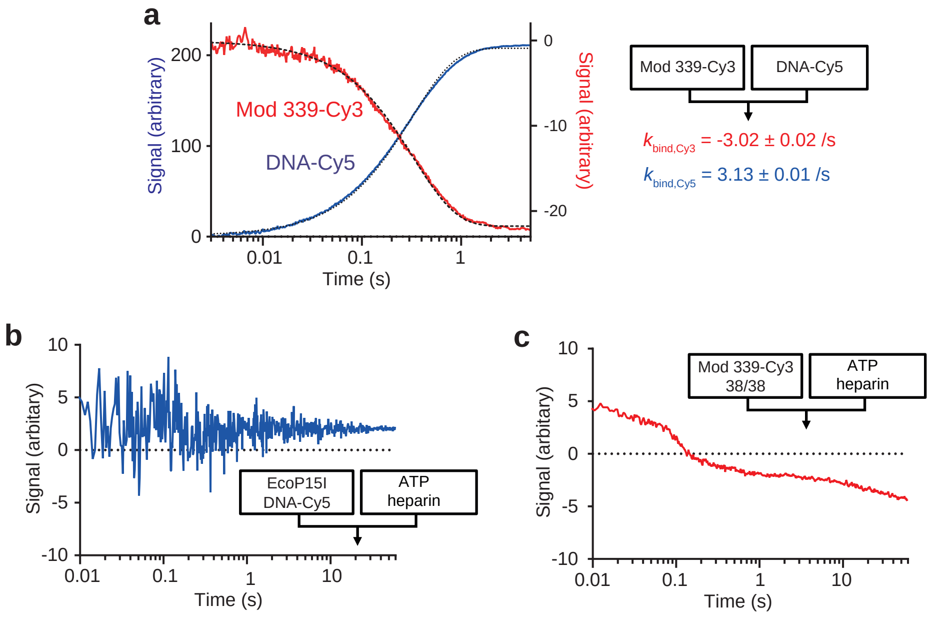

Supplementary Fig. 6: Fluorescence changes due to DNA binding and ATP-dependent dissociation of cyanine dye-labelled EcoP15I from DNA. a, Stopped flow fluorescence measurement of binding of 75 nM EcoP15I 339-Cy3 and 25 nM DNA-Cy5. Excitation wavelength was set to 546 nm and the emission of Cy3 (red, right *y*-axis) and Cy5 (blue, left *y*-axis) measured simultaneously. The dashed and dotted lines are single exponential fits to give the rate constants shown (errors S.E.M.). **b**, Stopped flow fluorescence measurement of the ATP-dependent dissociation of 75 nM unlabelled EcoP15I from 25 nM DNA-Cy5, with final concentrations of 4 mM ATP and 2.5 µM heparin trap. Excitation wavelength was set to 600 nm and the emission of Cy5 (blue) was measured. **c**, Stopped flow fluorescence measurement of the ATP-dependent dissociation of 75 nM EcoP15I 339-Cy3 from 25 nM unlabelled 38/38 oligoduplex (see Supplementary Fig. 13) , with final concentrations of 4 mM ATP and 2.5 µM heparin trap. Excitation wavelength was set to 546 nm and the emission of Cy3 (red) measured.

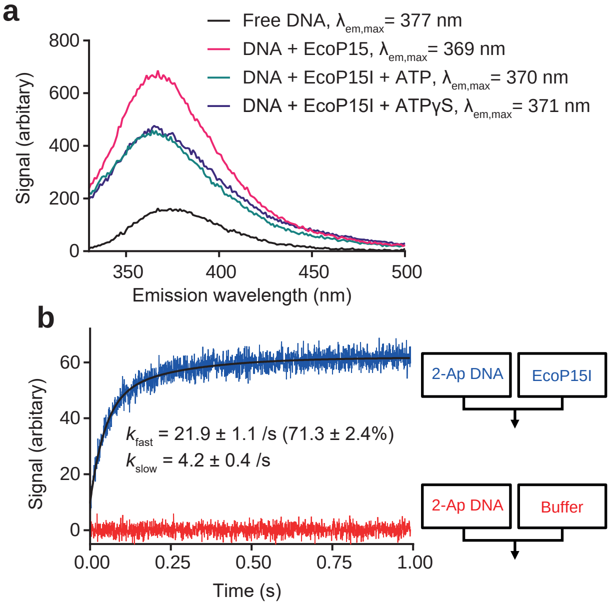

Supplementary Fig. 7: Fluorescence changes associated with EcoP15I association with a 2-aminopurine labelled oligoduplex. a, Steady-state 2-aminopurine fluorescence emission spectra for 500 nM 2-Ap oligoduplex (Fig. 2d) made by annealing 20/50_P15F_11Cy5 and 20/50_P15R (Supplementary Table 4) and, where indicated, 600 nM EcoP15I, 4 mM ATP and 4 mM ATPγS. Excitation wavelength was set to 311 nm. **b**, Stopped flow fluorescence measurement at 311 ± 3 nm and using a 360 nm long-pass filter of 125 nM 2-Ap oligoduplex mixed with either 150 nM EcoP15I or a buffer control. The association curve was fitted with a double exponential (solid black line) to give the parameters shown (errors S.E.M.).

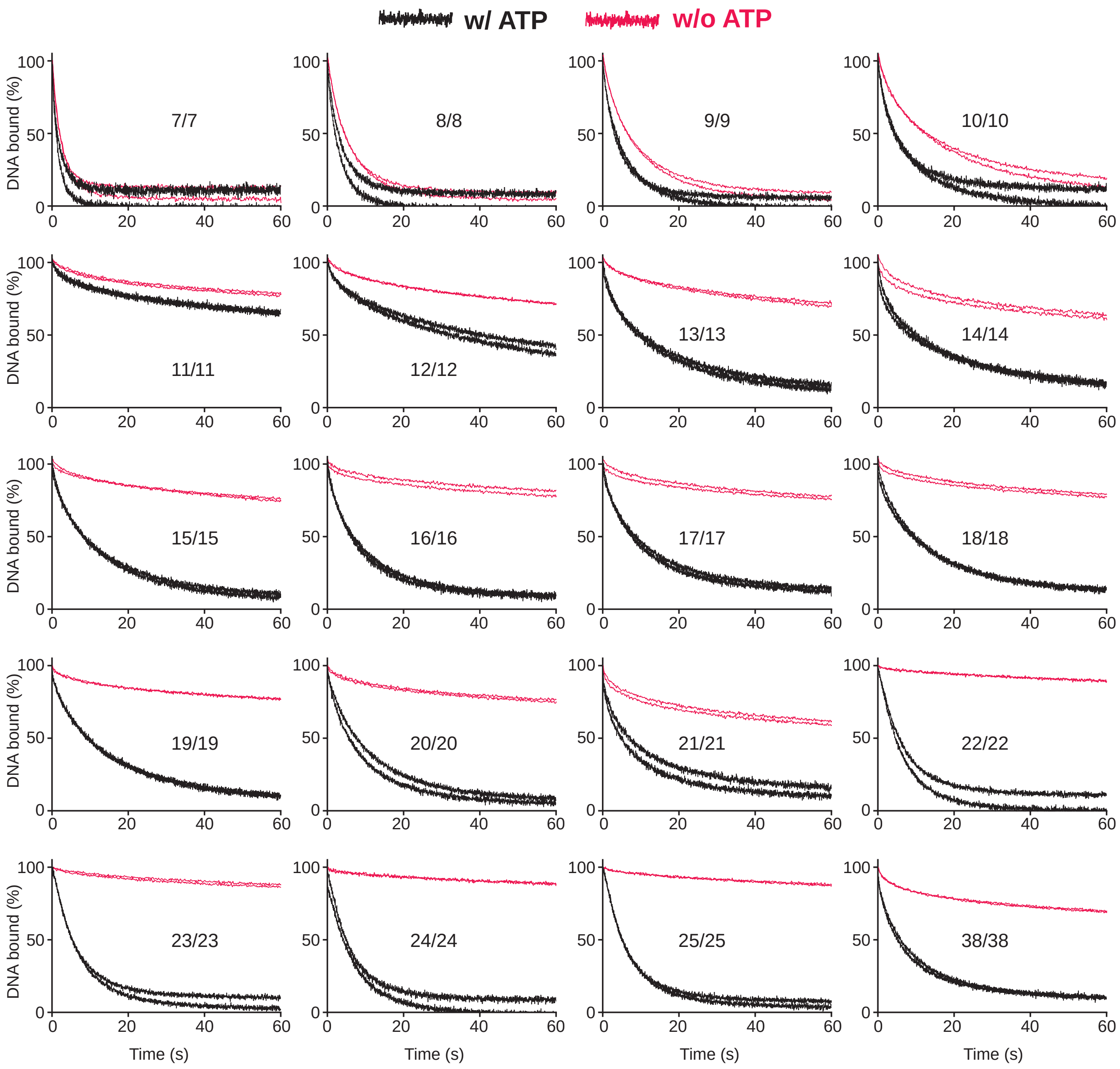

Supplementary Fig. 8: ATP-dependent DNA dissociation of EcoP15I as a function of downstream DNA length measured using stopped flow anisotropy. Release of prebound enzyme from its target site on oligoduplexes (named according to Supplementary Fig. 13) following mixing with heparin and with (black) or without (magenta) ATP, measured using stopped flow fluorescence anisotropy. Data from two independent repeats are shown.

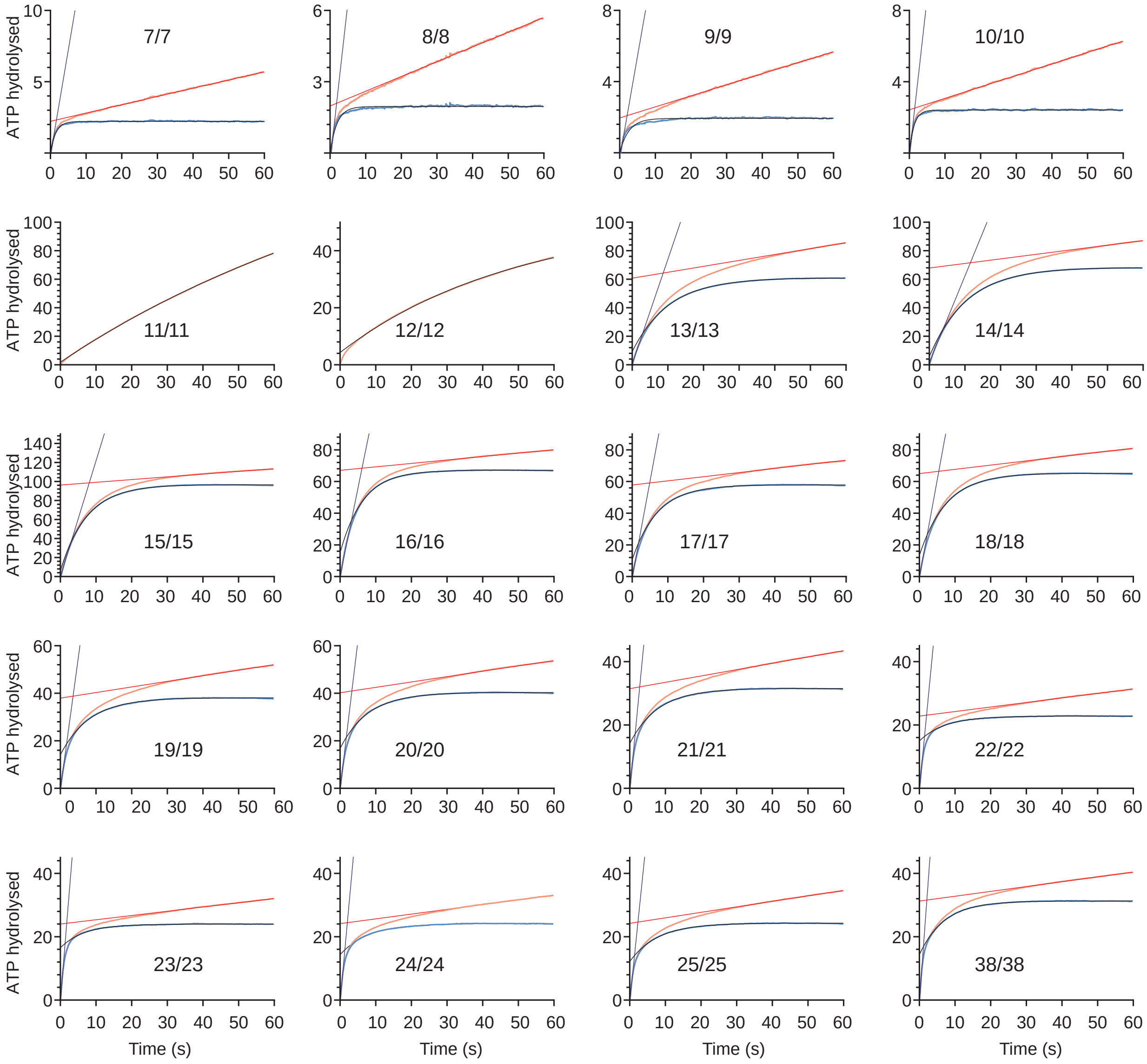

Supplementary Fig. 9: ATP hydrolysis by EcoP15I as a function of downstream DNA length measured using stopped flow fluorescence. Pre-bound EcoP15I and oligoduplexes (named according to Supplementary Fig. 13) were mixed with 4 mM ATP and the phosphate release measured using phosphate binding protein. Data was separated into different phases as in Toth et al (ref 31). The background steady-state rate of ATP hydrolysis due to heparin binding to free EcoP15I was estimated by linear regression (red line) to the raw data (light red) for t > 45.0 s. The raw data was corrected for the background ATPase to produce the DNA-dependent data (light blue) which was fitted for t > 3.5 s to $y = (A_2 \cdot (1 - \exp(-k_2 \cdot t))) + A_1$ (black line), to estimate the ATP amplitudes for the first (A_1) and second (A_2) ATPase bursts. k_2 was used as a measure of the lifetime of the second burst and $A_2 \cdot k_2$ as a measure of the ATPase rate of the second burst. The corrected data was fitted for t < 0.35 s by linear regression to estimate the ATPase rate of the first burst. The lifetime of the first burst was estimated by dividing A_1 by the ATPase rate. 11/11 and 12/12 use the data uncorrected for heparin as their off rate is low and so heparin binding effect is negligible and produce a first burst that was too small to be reliably fitted. 7/7 to 10/10 only give a first burst phase. Fitted values are shown in Supplementary Fig. 14. One representative dataset shown from two independent repeats.

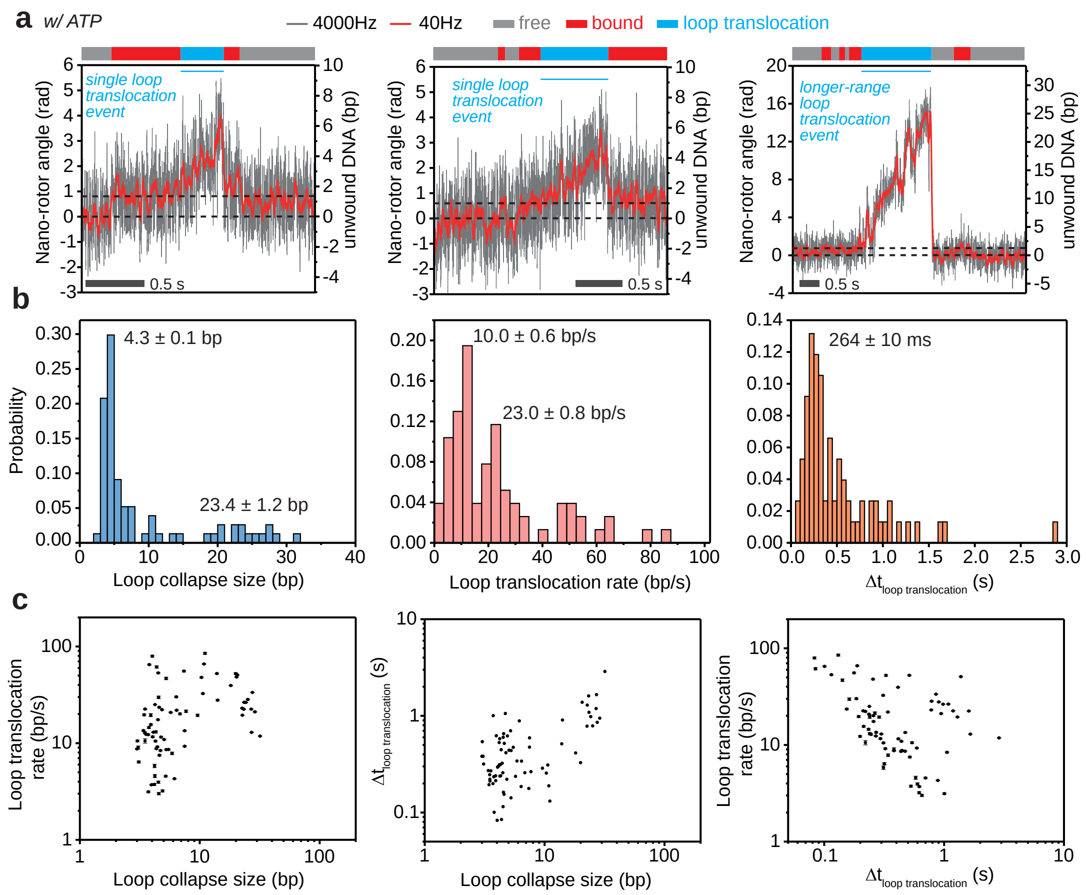

Supplementary Fig. 10: DNA loop translocation properties of wild type EcoP15I at 6 pN stretching force are similar to those obtained at 3 pN stretching force. a, Representative examples of different loop translocation events including color coded identification for different wild type EcoP15I-DNA interaction states (grey, free state; red, bound state; blue, loop translocation) at an applied external force of ~6 pN. **b**, Maximum loop size and loop translocation rate exhibiting bimodal distributions, with mean values of: 4.3 ± 0.1 bp, 23.4 ± 1.2 bp and 10.0 ± 0.6 bp/s, 23.0 ± 0.8 bp/s, respectively (error S.E.). The loop translocation time ($\Delta t_{\text{loop translocation}}$) exhibits a monomodal distribution with a mean value of 264 ± 10 ms (*N* = 77, error S.E.). **c**, Plots of the loop translocation rate vs. loop size (left), $\Delta t_{\text{loop translocation}}$ vs. loop size (middle) and the loop translocation rate vs. $\Delta t_{\text{loop translocation}}$ (right) plotted for individual events.

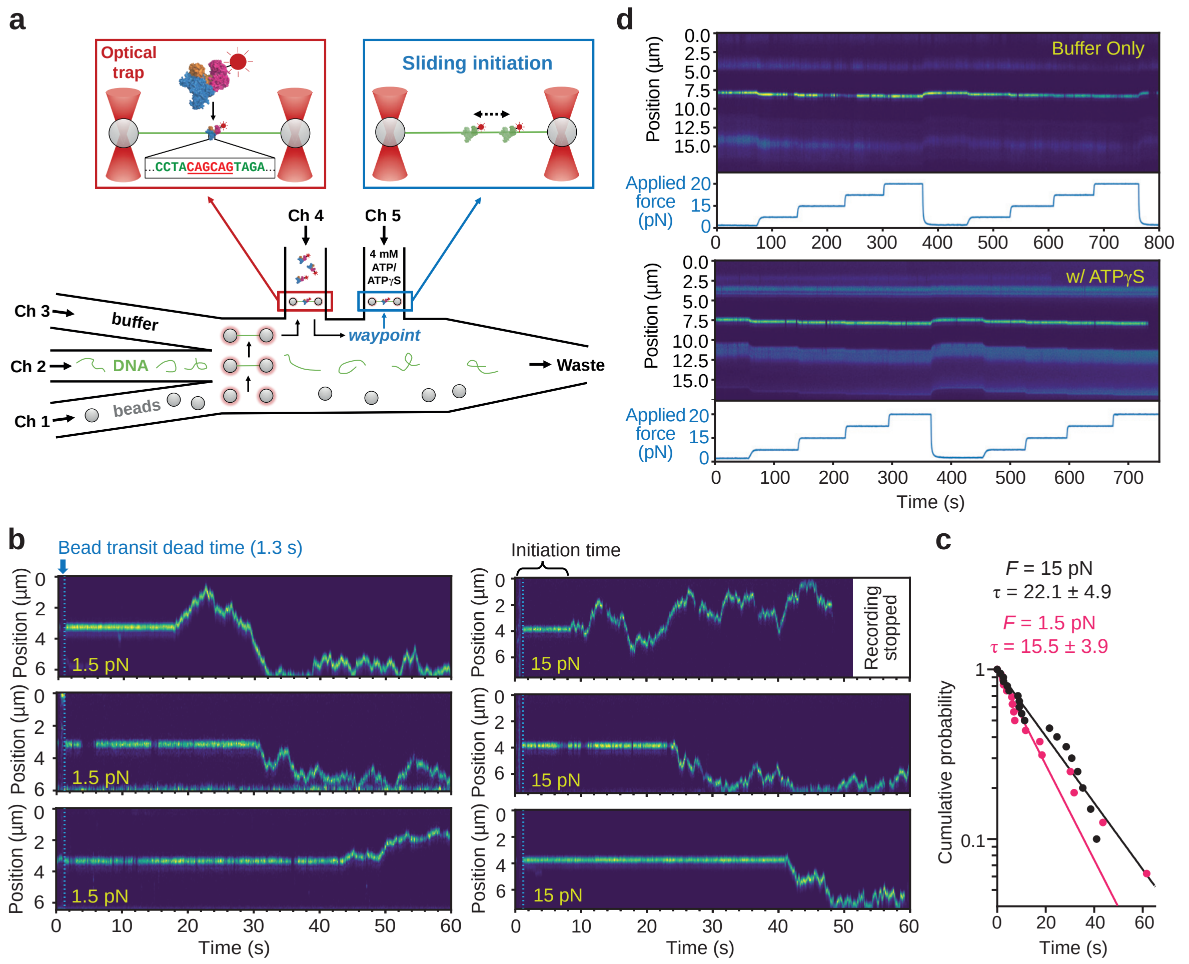

Supplementary Fig. 11: The effect of DNA stretching force on the initiation of sliding by EcoP15I. a, Schematic of the C-Trap flow cell. DNA tethers were formed in Channels 1-3 before binding labelled enzyme (655 nm quantum dot-streptavidin-biotinEcoP15I) to a single central recognition site in Channel 4. To initiate sliding, the tether was moved from a fixed waypoint in Channel 3 containing only imaging buffer to a waypoint in Channel 5 containing buffer plus 4 mM ATP. During this transit period the precise ATP concentration is undefined, and the tether is displaced from the kymograph axis by hydrodynamic drag such that enzyme position cannot be determined, resulting in a dead time of 1.3 s. **b**, Representative kymographs at either 1.5 or 15 pN showing an initial period where EcoP15I remains bound at the site, after which sliding initiates to produce 1-D diffusive motion. Zero time is defined as the start of the transit period, with the dotted line showing when the waypoint in Channel 5 is reached. Occasional gaps in the traces are due to quantum dot blinking. **c**, Sliding initiation times determined from kymographs (N = 16 for 1.5 pN and N = 20 for 15 pN) represented as inverted cumulative probability plots fitted to exponential functions to give the initiation time (τ) (Standard Error). Uncertainty due to the transit dead time is smaller than the diameter of data points. **d**, Representative kymographs showing the positions of site-bound EcoP15I with or without 4 mM ATPγS while changing the DNA stretching force (1.5, 5, 10, 15, and 20 pN) (N = 19 and 12 for Buffer Only and ATPγS, respectively). Bead autofluorescence is visible at the upper and lower kymograph edges. Occasional gaps in the traces are due to quantum dot blinking. In each case, EcoP15I remained bound at the central site throughout, and elevated forces (up to 50 pN) did not induce 1-D diffusion.

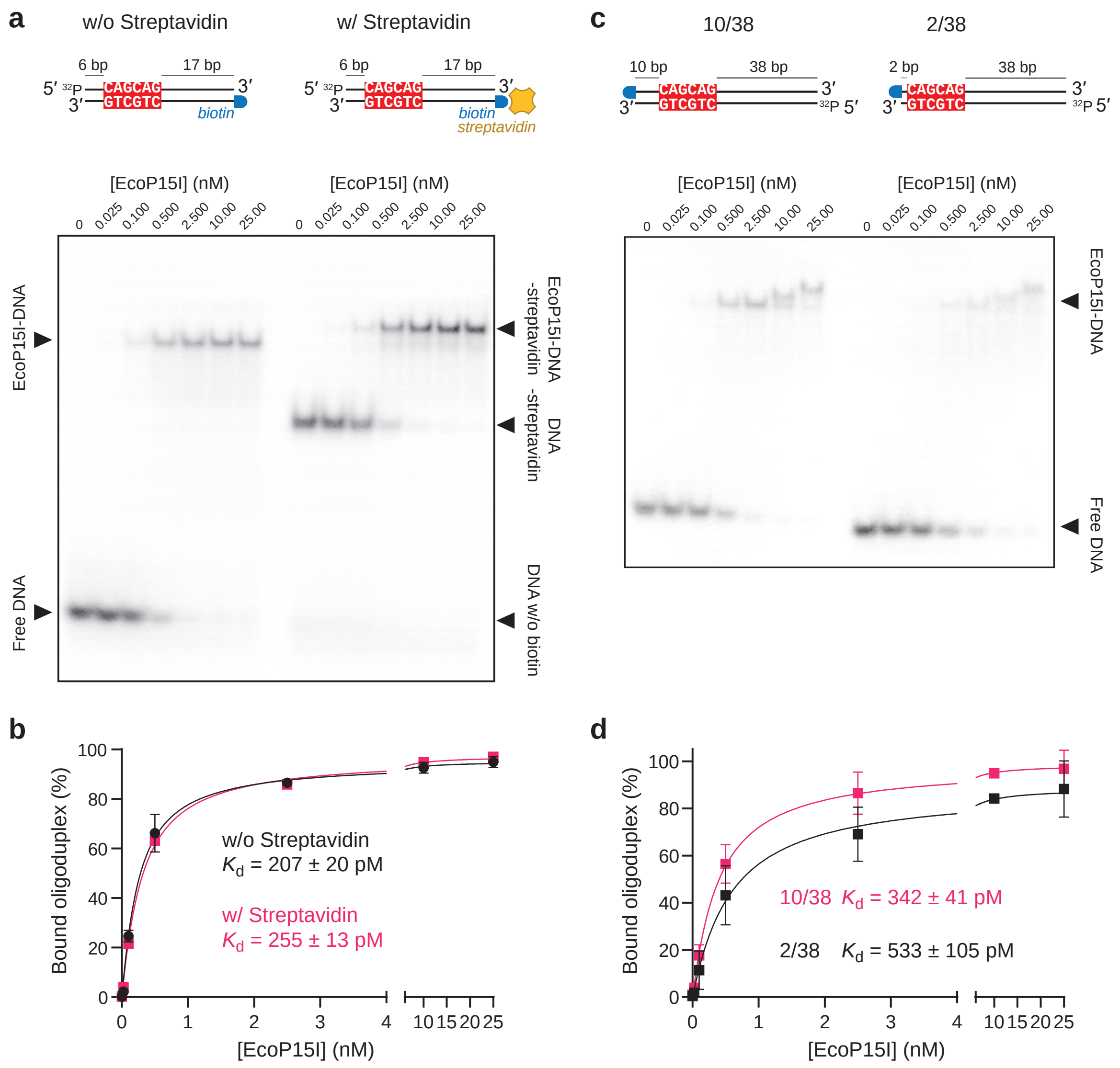

Supplementary Fig. 12: EcoP15I binding to oligoduplexes. a, Band-shift assays using 0.1 nM ^{32}P-labelled oligoduplex with 17 bp downstream DNA, without (w/o) or with (w/) pre-bound streptavidin mixed with varying concentrations of EcoP15I as indicated. The free and bound forms were separated by native polyacrylamide electrophoresis (representative gel of 3 repeats). **b**, The mean of the quantified data (*N* = 3, error bars S.D.) were fit using the tight binding equation (K_d, error S.E.). **c,** Band-shift assays using 0.1 nM ^{32}P-labelled oligoduplexes with either 10 bp (10/38) or 2 bp (2/38) upstream DNA mixed with varying concentrations of EcoP15I as indicated. The free and bound forms were separated by native polyacrylamide electrophoresis (representative gel of 3 repeats). **d**, The mean of the quantified data (*N* = 3, error bars S.D.) were fit using the tight binding equation (K_d, error S.E.).

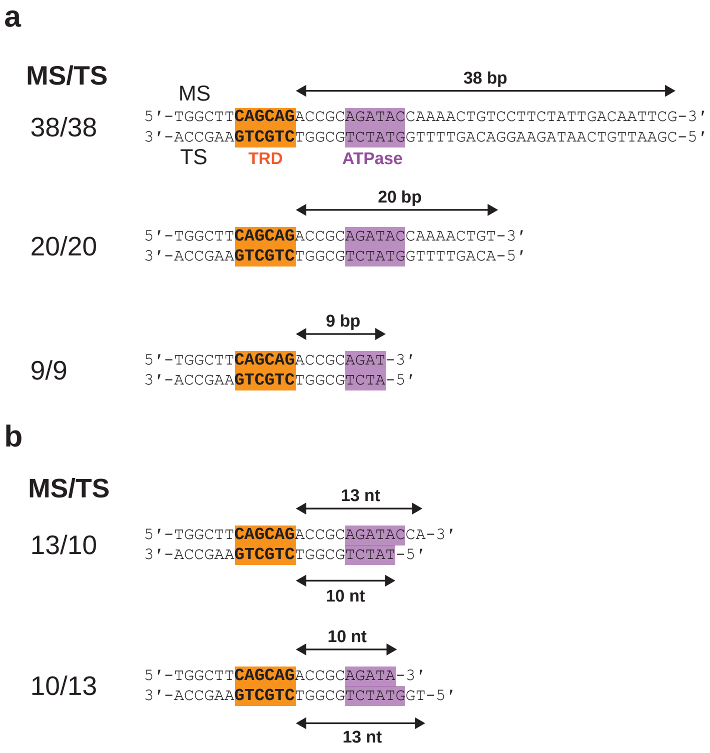

Supplementary Fig. 13: Examples of DNA substrate nomenclature. The DNA recognition site is highlighted in orange and the region contacted by the helicase domain is highlighted in pink. Each substrate contains six fixed residues of DNA upstream of the DNA recognition site and a variable number of base pairs downstream. The naming system is “X/Y”, where “X” and “Y” represent the lengths in nucleotides downstream of the target site of the MS and TS, respectively. **a**, Sequence of the 50 bp “full length” DNA substrate 38/38 on which EcoP15I is known to have normal activity and examples of substrates with truncated downstream dsDNA. **b**, Examples of substrates with 5′ or 3′ overhangs downstream of the DNA recognition site used to investigate the effect of strand polarity on EcoP15I activity.

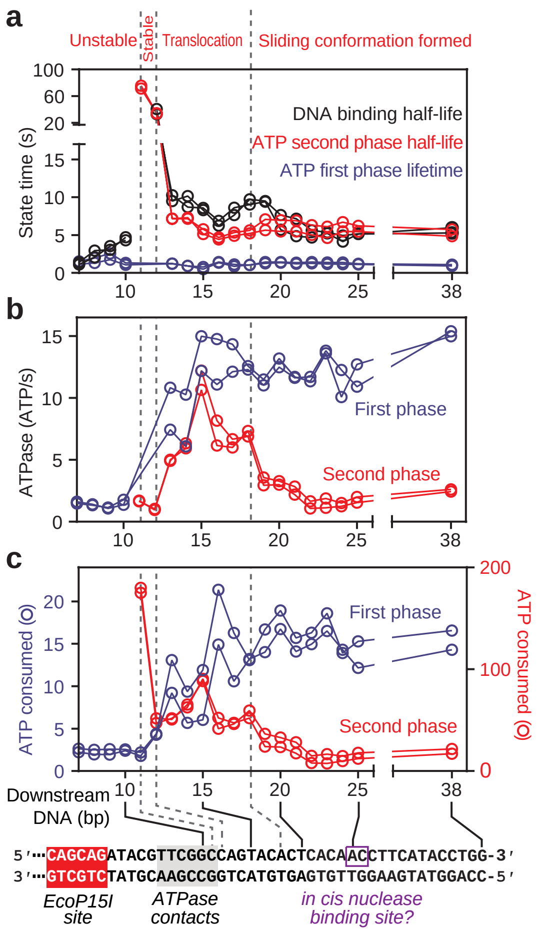

Supplementary Fig. 14: Downstream length-dependence of DNA dissociation and ATPase kinetics. Circles show fitted parameters from two repeat experiments (Supplementary Figs. 8, 9). Vertical dotted lines align to the downstream DNA sequence map below panel c. **a**, Lifetimes of the first (blue) and second (red) ATPase phases and of DNA dissociation with ATP (black). Second phases were not observed for 7/7 – 9/9. Only an uncoupled second phase was observed for 11/11 and 12/12. **b**, Rates of ATP hydrolysis of the first (blue) and second (red) phases. **c**, Number of ATPs consumed in the first (blue, lefthand *y*-axis) and second (red, righthand *y*-axis) ATPase phases.

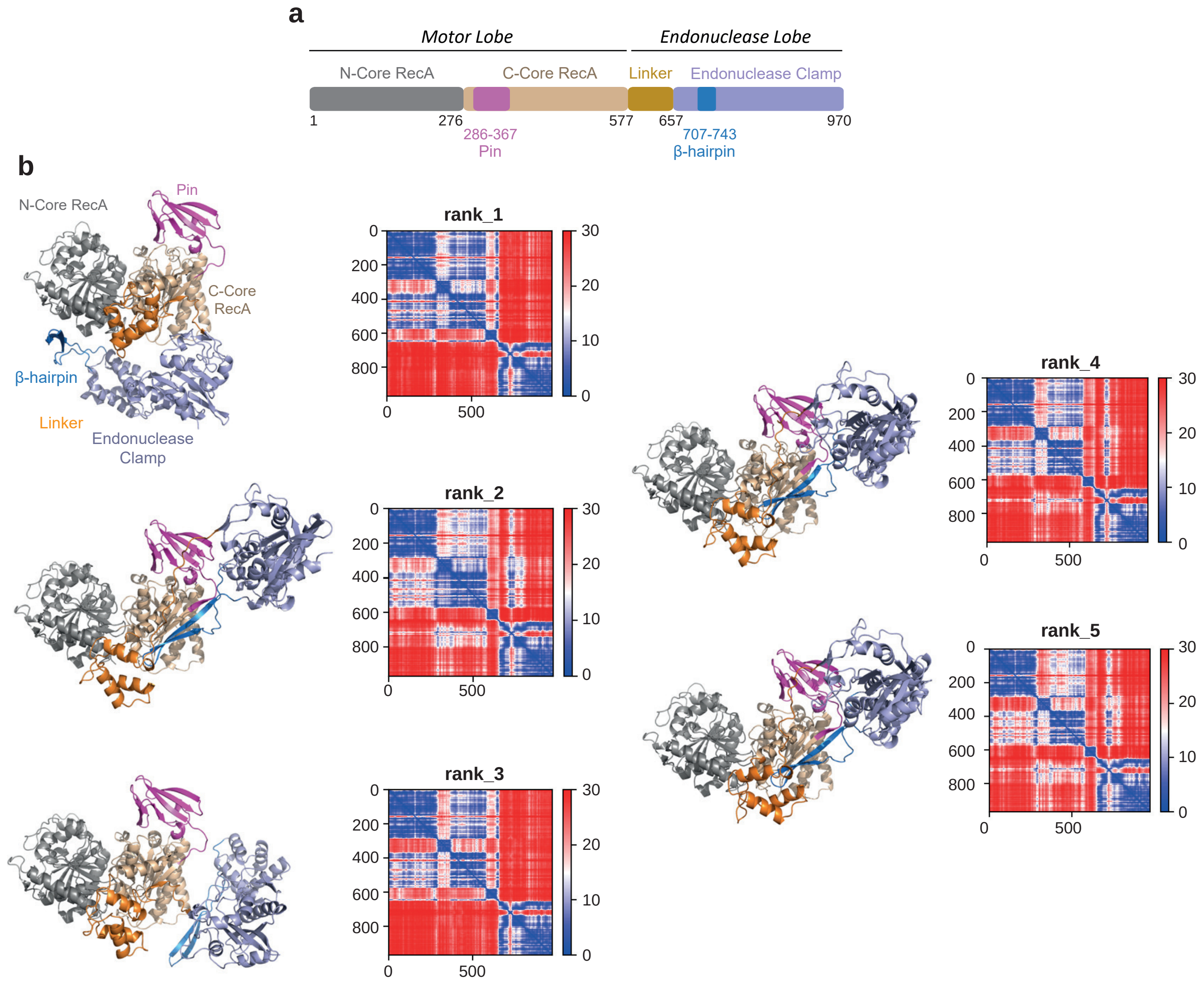

Supplementary Fig. 15: Predicted Aligned Error (PAE) of ranked AlphaFold 2 structures of EcoP15I Res. a, Domain arrangement of Res determined from crystal structure[15] and AlphaFold 2 structural predictions. **b,** AlphaFold 2 structural predictions and PAEs. In the structures, domains are colored as indicated top left and panel a. In all structures, the PAEs indicate that AlphaFold 2 predicts well-defined relative positions and orientations for residues of the helicase subdomains, the Pin, the α-helical bundle of the linker and the core Endonuclease Clamp[48]. The relative orientations of the N-core and C-core RecA folds are also well defined, particularly for rank_1, but the Pin has a less well-defined relative position that might reflect necessity for motion during translocation[15]. The nuclease lobe is independent of the helicase lobe, suggesting relative flexibility that might explain the absence of this lobe in the crystal structure and might be necessary to allow binding to the DNA cleavage site. The PAE for the Linker and β-hairpin indicate flexibility relative to the nuclease core. In rank_1 and rank_3, there are interactions between the Linker and both RecA cores. In rank_2, rank_4 and rank_5, the β-hairpin forms a pseudo-continuous β-sheet with the C-core RecA fold.

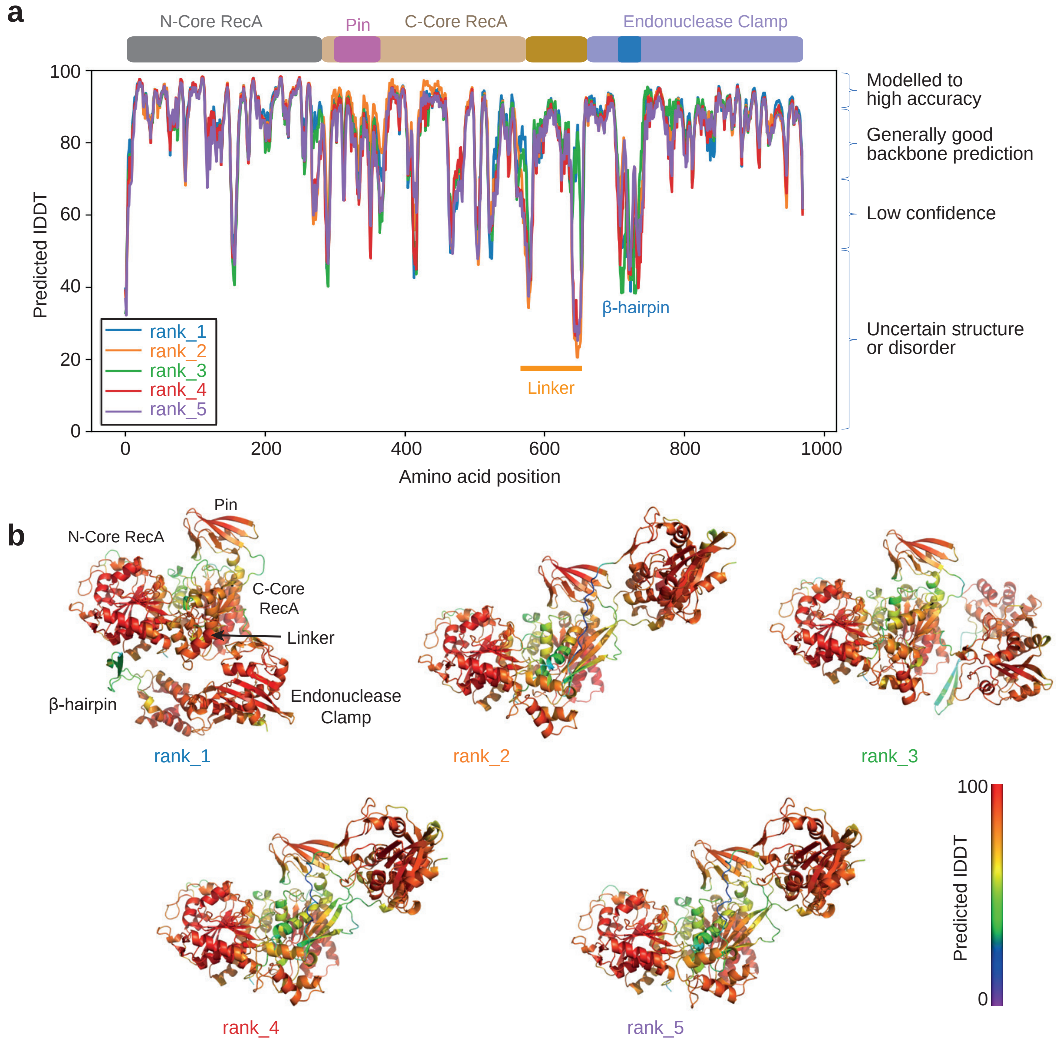

Supplementary Fig. 16: Predicted local distance difference test (pLDDT) of ranked AlphaFold 2 structures of EcoP15I Res. a, Plot of pLDDT (corresponding to each model's predicted score on the lDDT-Cα metric) against residue number for each ranked structure. The domains are indicated in the cartoon and labelled on the plot. The reliability interpretations of the pLDDT scores are indicated on the right. A pLDDT < 50 is a reasonably strong predictor of disorder[48] and corresponds to loops within the RecA and Pin folds, to the boundaries of the Linker and to the β-hairpin. **b,** Cartoon views of the ranked structures colored according to the predicted pLDDT score (key, right). The domains are labelled for the rank_1 structure and the structures are aligned to the N-Core RecA.

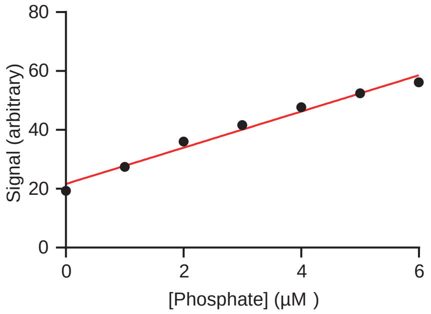

Supplementary Fig. 17: Conversion of raw fluorescence signal into phosphate released. A calibration plot of the raw fluorescence signal in the stopped flow at fixed concentrations of inorganic phosphate using a final concentration of 8 μM PBP. The slope of the linear regression was used to convert the observed signal to released phosphate and hence ATPs hydrolysed.

Name	5′-3′ sequence
EcoP15I target I	TCAGC**CAGCAG**ACCGCAGATACCAAAACTGTCCTTCTATTGACAATTCGG
EcoP15I target II	GGCCCCGAATTGTCAATAGAAGGACAGTTTTGGTATCTGCGGTCTGCTGGCTGAGGGACACT
EcoP15I no-target I	TCAGCGCTTATGCGTGGGAGGCCATTGATATAGGTATATTC
EcoP15I no-target II	GGCCGAATATACCTATATCAATGGCCTCCCACGCATAAGCGCTGAGGGACACT
DNA spacer primer I	GCAGCCACTAGTTGCCATAGATTATAGCTAAGG
DNA spacer primer II	CTCCCTCAGCAGGAGAACGAGGATATT

Supplementary Table 1: Oligodeoxyribonucleotide sequences used for EcoP15I target sequence, no-target sequence and PCR for dsDNA spacer. The site for EcoP15I (CAGCAG) is underlined and in bold font. Oligodeoxyribonucleotides synthesised by Sigma Aldrich.

Name		Downstream Length	5′-3′ Sequence
Slidef10	Variable primers	10 bp	CCGAACGTATCTGCTGTAGT
SlideF11		11 bp	GCCGAACGTATCTGCTGTAG
SlideF12		12 bp	GGCCGAACGTATCTGCT
SlideF13		13 bp	TGGCCGAACGTATCTGCT
SlideF14		14 bp	CTGGCCGAACGTATCTGCT
SlideF15		15 bp	ACTGGCCGAACGTATCTGC
SlideF16		16 bp	TACTGGCCGAACGTATCTGC
SlideF17		17 bp	GTACTGGCCGAACGTATCTG
SlideF18		18 bp	TGTACTGGCCGAACGTATCT
SlideF19		19 bp	GTGTACTGGCCGAACGTATCT
SlideF20		20 bp	AGTGTACTGGCCGAACGTATC
SlideF21		21 bp	GAGTGTACTGGCCGAACGTAT
SlideF22		22 bp	TGAGTGTACTGGCCGAACG
SlideF23		23 bp	GTGAGTGTACTGGCCGAACG
SlideF24		24 bp	TGTGAGTGTACTGGCCGAAC
SlideF25		25 bp	TTGTGAGTGTACTGGCCGAAC
SlideF26		26 bp	GTTGTGAGTGTACTGGCCGAA
slideF27		27 bp	GGTTGTGAGTGTACTGGCCGA
SlideF28		28 bp	AGGTTGTGAGTGTACTGGCCGA
SlideF29		29 bp	AAGGTTGTGAGTGTACTGGCCGA
SlideF30		30 bp	GAAGGTTGTGAGTGTACTGGCCGA
SlideF31		31 bp	TGAAGGTTGTGAGTGTACTGG
SlideF32		32 bp	ATGAAGGTTGTGAGTGTACTGG
slideF33		33 bp	TATGAAGGTTGTGAGTGTACTGG
slideF34		34 bp	GTATGAAGGTTGTGAGTGTACTGG
SlideF35		35 bp	GGTATGAAGGTTGTGAGTGTACTG
SlideF36		36 bp	AGGTATGAAGGTTGTGAGTGTACTG
SlideF37		37 bp	CAGGTATGAAGGTTGTGAGTGT
SlideF38		38 bp	CCAGGTATGAAGGTTGTGAG
SlideRbio	constant primer	N/A	TCTAGATCACACCTCTTCCTTC

Supplementary Table 2: Primer sequences used for PCR to make substrates in Fig. 3c-e. Oligodeoxyribonucleotides synthesised by Integrated DNA Technologies.

Tail-to-tail EcoP15I & EcoPI DNA	TCTAGATCACACCTCTTCCTTCAACAATTTCTT**<u>CTTGATG</u>**TGCTCCGCCAAGATACGGCTGAAA<br>AGGGGCGGAACAGCATTACCCA**<u>CTTGATG</u>**TCCTTGTTGGCGACGGTTGCCCTTAAAGATCAT<br>CCAATCAGGGAATGTCTGAATGCGGGCGGCCTCGCGAACTGTAAGAGAGCGGTTTAAGATC<br>GGGTGGATAGGGAAAGCATCGTTACCGGGAACCATGGTTAATGCGGTACAGTTCTGGGGGA<br>AGATCGTTTTCGGGTAAACGCCCGCCTTCTGGAATCAGCTTATAGCGTTCGATGATCTTTTCC<br>GTGTGCTTAAGGGGGACGTGATTGAAGTCCTTATCCTCCTTAATTTCAACTAAGTC**<u>CTGGATG</u>**<br>GCATACCAACAGTTCTCCCAAGAAAATGTCCCATCTTCTCCGTTTTCTGAATGGGTTGGCAAT<br>GGGAAATTGACTTCATGCCCCAGACGGTTTCCAATGATAAAGACACGTTCGCGCAACTGAGG<br>CACACCATAATCAGCCATATTCAGAAGCTTTAATTTGTAGTCATAGCCTGTTTTATCAAGTTCC<br>TTATAAACACCTTTGATAAAATCACCTTTATCCATAGTCAGAAGCCCCTTGACATTCTCAATCA<br>CGAAGTATTTCGGCTTCAACTGATTCAAAATGCGGATATATTCTAAGATTAACTTATTGCGTTC<br>ATCTTCTGCGGTGCGACGTTCGAAGTTCGACGATACAC**<u>GGTCT</u>**CCGATAGTTGAGAACCCCT<br>GACAGGGAGGTCCTCCGATTAACACGTCGATCTCTTCTCCTTCGATTAAATTAAATAATTCAA<br>AACCTGTCAACTGATTAATGTCGTCGCTAAAGAAAGGGATCTCGGGGAAATTGAATTCGTGT<br>GTTTTCGCGACAATAGGATTCGAGTCTACAGCCAGTTTAATTTCGAAGCCGGCTTCTTGGAAG<br>CCCAGGTGCATACTA**<u>CAGCAG</u>**ATACGTTCGGCCAGTACACTCACAACCTTCATACCTGG

Supplementary Table 3: Sequence of dsDNA fragment used as a PCR template to make the substrate in Fig. 3 and Extended Data Fig. 4. Sites for EcoP15I (CAGCAG), EcoPI (AGACC) and LlaGI (CTNGAYG) are underlined and in bold font. DNA fragment synthesised by Integrated DNA Technologies.

Name	X = fluorophore	5′-3′ Sequence
2AP_38_Fwd	X = 2-aminopurine	TGGCTTCAGC**X**GACCGCAGATACCAAAACTGTCCTTCTATTGACAATTCG
38_Rev		CGAATTGTCAATAGAAGGACAGTTTTGGTATCTGCGGTCTGCTGAAGCCA
20/50_P15F_11Cy5	X = Cy5	GCCAGTGAA**X**TAACTGGCTTCAGCAGACCGCAGATACCAAAACTGTCCTT
20/50_P15R		AAGGACAGTTTTGGTATCTGCGGTCTGCTGAAGCCAGTTAATTCACTGGC

Supplementary Table 4: Internally fluorescent labelled oligodeoxyribonucleotide sequences and complementary strands annealed to make substrates for stopped-flow spectroscopy. Oligodeoxyribonucleotides synthesised by Integrated DNA Technologies (2-aminopurine) or Eurofins (Cyanine 5).

Name	5′-3′ sequence
38_Fwd	TGGCTTCAGCAGACCGCAGATACCAAAACTGTCCTTCTATTGACAATTCG
25_Fwd	TGGCTTCAGCAGACCGCAGATACCAAAACTGTCCTTC
24_Fwd	TGGCTTCAGCAGACCGCAGATACCAAAACTGTCCTT
23_Fwd	TGGCTTCAGCAGACCGCAGATACCAAAACTGTCCT
22_Fwd	TGGCTTCAGCAGACCGCAGATACCAAAACTGTCC
21_Fwd	TGGCTTCAGCAGACCGCAGATACCAAAACTGTC
20_Fwd	TGGCTTCAGCAGACCGCAGATACCAAAACTGT
19_Fwd	TGGCTTCAGCAGACCGCAGATACCAAAACTG
18_Fwd	TGGCTTCAGCAGACCGCAGATACCAAAACT
17_Fwd	TGGCTTCAGCAGACCGCAGATACCAAAAC
16_Fwd	TGGCTTCAGCAGACCGCAGATACCAAAA
15_Fwd	TGGCTTCAGCAGACCGCAGATACCAAA
14_Fwd	TGGCTTCAGCAGACCGCAGATACCAA
13_Fwd	TGGCTTCAGCAGACCGCAGATACCA
12_Fwd	TGGCTTCAGCAGACCGCAGATACC
11_Fwd	TGGCTTCAGCAGACCGCAGATAC
10_Fwd	TGGCTTCAGCAGACCGCAGATA
9_Fwd	TGGCTTCAGCAGACCGCAGAT
8_Fwd	TGGCTTCAGCAGACCGCAGA
7_Fwd	TGGCTTCAGCAGACCGCAG
38_Rev	CGAATTGTCAATAGAAGGACAGTTTTGGTATCTGCGGTCTGCTGAAGCCA
25_Rev	GAAGGACAGTTTTGGTATCTGCGGTCTGCTGAAGCCA
24_Rev	AAGGACAGTTTTGGTATCTGCGGTCTGCTGAAGCCA
23_Rev	AGGACAGTTTTGGTATCTGCGGTCTGCTGAAGCCA
22_Rev	GGACAGTTTTGGTATCTGCGGTCTGCTGAAGCCA
21_Rev	GACAGTTTTGGTATCTGCGGTCTGCTGAAGCCA
20_Rev	ACAGTTTTGGTATCTGCGGTCTGCTGAAGCCA
19_Rev	CAGTTTTGGTATCTGCGGTCTGCTGAAGCCA
18_Rev	AGTTTTGGTATCTGCGGTCTGCTGAAGCCA
17_Rev	GTTTTGGTATCTGCGGTCTGCTGAAGCCA
16_Rev (12+4T_Rev)	TTTTGGTATCTGCGGTCTGCTGAAGCCA
15_Rev (12+3T_Rev)	TTTGGTATCTGCGGTCTGCTGAAGCCA
14_Rev (12+2T_Rev)	TTGGTATCTGCGGTCTGCTGAAGCCA
13_Rev (12+1T_Rev)	TGGTATCTGCGGTCTGCTGAAGCCA
12_Rev	GGTATCTGCGGTCTGCTGAAGCCA
11_Rev	GTATCTGCGGTCTGCTGAAGCCA
10_Rev	TATCTGCGGTCTGCTGAAGCCA
9_Rev	ATCTGCGGTCTGCTGAAGCCA
8_Rev	TCTGCGGTCTGCTGAAGCCA
7_Rev	CTGCGGTCTGCTGAAGCCA
12+5T_Rev	TTTTTGGTATCTGCGGTCTGCTGAAGCCA
N35_Fwd_Phos	PO_3^--GCAGATACCAAAACTGTCCTTCTATTGACAATTCG
N23_Rev	CGAATTGTCAATAGAAGGACAGTTTTGGTATCTGC
N15_Rev_Phos	PO_3^--GGTCTGCTGAAGCCA

Supplementary Table 5: Oligodeoxyribonucleotide sequences annealed to make substrates for stopped-flow spectroscopy. Oligodeoxyribonucleotides synthesised by Integrated DNA Technologies.

Name	5′-3′ sequence
38_Fwd_Hex	HEX-5′-TGGCTTCAGCAGACCGCAGATACCAAAACTGTCCTTCTATTGACAATTCG-3′
25_Fwd_Hex	HEX-5′-TGGCTTCAGCAGACCGCAGATACCAAAACTGTCCTTC-3′
24_Fwd_Hex	HEX-5′-TGGCTTCAGCAGACCGCAGATACCAAAACTGTCCTT-3′
23_Fwd_Hex	HEX-5′-TGGCTTCAGCAGACCGCAGATACCAAAACTGTCCT-3′
22_Fwd_Hex	HEX-5′-TGGCTTCAGCAGACCGCAGATACCAAAACTGTCC-3′
21_Fwd_Hex	HEX-5′-TGGCTTCAGCAGACCGCAGATACCAAAACTGTC-3′
20_Fwd_Hex	HEX-5′-TGGCTTCAGCAGACCGCAGATACCAAAACTGT-3′
19_Fwd_Hex	HEX-5′-TGGCTTCAGCAGACCGCAGATACCAAAACTG-3′
18_Fwd_Hex	HEX-5′-TGGCTTCAGCAGACCGCAGATACCAAAACT-3′
17_Fwd_Hex	HEX-5′-TGGCTTCAGCAGACCGCAGATACCAAAAC-3′
16_Fwd_Hex	HEX-5′-TGGCTTCAGCAGACCGCAGATACCAAAA-3′
15_Fwd_Hex	HEX-5′-TGGCTTCAGCAGACCGCAGATACCAAA-3′
14_Fwd_Hex	HEX-5′-TGGCTTCAGCAGACCGCAGATACCAA-3′
13_Fwd_Hex	HEX-5′-TGGCTTCAGCAGACCGCAGATACCA-3′
12_Fwd_Hex	HEX-5′-TGGCTTCAGCAGACCGCAGATACC-3′
11_Fwd_Hex	HEX-5′-TGGCTTCAGCAGACCGCAGATAC-3′
10_Fwd_Hex	HEX-5′-TGGCTTCAGCAGACCGCAGATA-3′
10_Phos_Fwd_Hex	HEX-5′-TGGCTTCAGCAGACCGCAGATA-3′-PO_3^-
9_Fwd_Hex	HEX-5′-TGGCTTCAGCAGACCGCAGAT-3′
8_Fwd_Hex	HEX-5′-TGGCTTCAGCAGACCGCAGA-3′
7_Fwd_Hex	HEX-5′-TGGCTTCAGCAGACCGCAG-3′
12+1T_Fwd_Hex	HEX-5′-TGGCTTCAGCAGACCGCAGATACCT-3′
12+2T_Fwd_Hex	HEX-5′-TGGCTTCAGCAGACCGCAGATACCTT-3′
12+3T_Fwd_Hex	HEX-5′-TGGCTTCAGCAGACCGCAGATACCTTT-3′
12+4T_Fwd_Hex	HEX-5′-TGGCTTCAGCAGACCGCAGATACCTTTT-3′
12+5T_Fwd_Hex	HEX-5′-TGGCTTCAGCAGACCGCAGATACCTTTTT-3′
Nick3_Fwd_Hex	HEX-5′-TGGCTTCAGCAGACC-3′

Supplementary Table 6: Hexachlorofluoroscein-labelled oligodeoxyribonucleotide sequences annealed to make substrates for stopped-flow spectroscopy. Oligodeoxyribonucleotides synthesised by Integrated DNA Technologies.
