# Supplementary material for: Short-range translocation by a restriction enzyme motor triggers diffusion along DNA
Source: Nat Chem Biol. 2024 Jan 2;20(6):689–98. doi: 10.1038/s41589-023-01504-1 (PMC11142916; doi:10.1038/s41589-023-01504-1)
Supplement: Supplementary file 2 — Reporting Summary [file 41589_2023_1504_MOESM2_ESM.pdf]

nature research

Corresponding author(s): Mark Szczelkun, Ralf Seidel

Last updated by author(s): 29/09/2023

Reporting Summary

Nature Research wishes to improve the reproducibility of the work that we publish. This form provides structure for consistency and transparency in reporting. For further information on Nature Research policies, see our Editorial Policies and the Editorial Policy Checklist.

Statistics

For all statistical analyses, confirm that the following items are present in the figure legend, table legend, main text, or Methods section.

n/a	Confirmed	
☐	☒	The exact sample size (*n*) for each experimental group/condition, given as a discrete number and unit of measurement
☒	☐	A statement on whether measurements were taken from distinct samples or whether the same sample was measured repeatedly
☐	☒	The statistical test(s) used AND whether they are one- or two-sided<br>*Only common tests should be described solely by name; describe more complex techniques in the Methods section.*
☒	☐	A description of all covariates tested
☐	☒	A description of any assumptions or corrections, such as tests of normality and adjustment for multiple comparisons
☐	☒	A full description of the statistical parameters including central tendency (e.g. means) or other basic estimates (e.g. regression coefficient) AND variation (e.g. standard deviation) or associated estimates of uncertainty (e.g. confidence intervals)
☐	☒	For null hypothesis testing, the test statistic (e.g. *F*, *t*, *r*) with confidence intervals, effect sizes, degrees of freedom and *P* value noted<br>*Give P values as exact values whenever suitable.*
☒	☐	For Bayesian analysis, information on the choice of priors and Markov chain Monte Carlo settings
☒	☐	For hierarchical and complex designs, identification of the appropriate level for tests and full reporting of outcomes
☒	☐	Estimates of effect sizes (e.g. Cohen's *d*, Pearson's *r*), indicating how they were calculated

Our web collection on statistics for biologists contains articles on many of the points above.

Software and code

Policy information about availability of computer code

Data collection	Magnetic tweezers single molecule data were collected using Labview 2016 and CUDA software as published in Huhle et al, Nat Commun, 2015. Stopped flow data were collected using Kinetic Studio 5.1.0.6 (TgK Scientific). Lumicks single molecule optical tweezers data was collected using LUMICKS Pylake available at https://github.com/lumicks/pylake (DOI 10.5281/zenodo.7945384).<br>Steady-state fluorescence was collected using Cary Eclipse Scan software [v1.1(132)].
Data analysis	Magnetic tweezers single molecule data were analysed as described in Kauert et al, Nat Struct Mol Biol, 2023<br>Lumicks single molecule optical tweezers data was analysed using LUMICKS Pylake available at https://github.com/lumicks/pylake (DOI 10.5281/zenodo.7945384).<br>State identification was performed using the vbFRET algorithm by Bronson et al, Biophys J ,1999<br>Gel data was quantified using Image Quant version TL (Cytiva)<br>DNA cleavage and stopped flow data were analysed using GraphPad Prism v9.4.0<br>Alphafold 2 protein prediction was performed at https://colab.research.google.com/github/sokrypton/ColabFold/blob/main/AlphaFold2.ipynb<br>Data fitting by ordinary differential equations was performed using Berkeley Madonna V.8.3.18

For manuscripts utilizing custom algorithms or software that are central to the research but not yet described in published literature, software must be made available to editors and reviewers. We strongly encourage code deposition in a community repository (e.g. GitHub). See the Nature Research guidelines for submitting code & software for further information.

Data

Policy information about availability of data

All manuscripts must include a data availability statement. This statement should provide the following information, where applicable:

- Accession codes, unique identifiers, or web links for publicly available datasets
- A list of figures that have associated raw data
- A description of any restrictions on data availability

Example data for the single molecule magnetic tweezers and ensemble assays are presented within the paper. The full datasets that support the findings of this study will be deposited at the data.bris research data repository. The data will be issued a unique British Library DataCite DOI (Digital Object Identifier) and maintained indefinitely. Published data cannot be changed.
Protein and DNA structure PDB files (PDB: 4ZCF15 and PDB:1BNA were obtained from the RCSB Protein Data Bank (https://www.rcsb.org/)

Field-specific reporting

Please select the one below that is the best fit for your research. If you are not sure, read the appropriate sections before making your selection.

☒ Life sciences ☐ Behavioural & social sciences ☐ Ecological, evolutionary & environmental sciences

For a reference copy of the document with all sections, see nature.com/documents/nr-reporting-summary-flat.pdf

Life sciences study design

All studies must disclose on these points even when the disclosure is negative.

Sample size	For the single molecule magnetic tweezers assay, we analysed >10 EcoP15I translocation events per DNA molecule and repeated the experiment 4 times (replication, below), resulting in ~130 single events. For the DNA cleavage assays, these are ensemble assays that were repeated 2-3 times (replication, below). For Lumicks optical tweezers experiments, 12-20 DNAs were analysed. Sample sizes were chosen to show experimental variation.
Data exclusions	data was not excluded for the single molecule and ensemble assays.
Replication	For the single molecule magnetic tweezers assay, the experiments were repeated with at least 4 separate DNA molecules. For the ensemble DNA cleavage assays, the experiments were repeated independently 3 times. For the stopped flow assays, the experiments were repeated independently 2-3 times. All attempts at independent replication were successful.
Randomization	Randomization was not relevant to this study as the measurement procedure entailed the unbiased and direct observation of stocahstic biological processes.
Blinding	Experiments were not blinded as the data analysis did not require subjective judgment or interpretation.

Reporting for specific materials, systems and methods

We require information from authors about some types of materials, experimental systems and methods used in many studies. Here, indicate whether each material, system or method listed is relevant to your study. If you are not sure if a list item applies to your research, read the appropriate section before selecting a response.

Materials & experimental systems

n/a	Involved in the study	
☒	☐	Antibodies
☒	☐	Eukaryotic cell lines
☒	☐	Palaeontology and archaeology
☒	☐	Animals and other organisms
☒	☐	Human research participants
☒	☐	Clinical data
☒	☐	Dual use research of concern

Methods

n/a	Involved in the study	
☒	☐	ChIP-seq
☒	☐	Flow cytometry
☒	☐	MRI-based neuroimaging
